# Contrasting response of microeukaryotic and bacterial communities to the interplay of seasonality and local stressors in shallow soda lakes

**DOI:** 10.1093/femsec/fiad095

**Published:** 2023-08-16

**Authors:** Zsuzsanna Márton, Bianka Csitári, Tamás Felföldi, András Hidas, Ferenc Jordán, Attila Szabó, Anna J Székely

**Affiliations:** Institute of Aquatic Ecology, Centre for Ecological Research, H-1113 Budapest, Hungary; National Multidisciplinary Laboratory for Climate Change, Centre for Ecological Research, H-1113 Budapest, Hungary; Doctoral School of Environmental Sciences, Eötvös Loránd University, H-1117 Budapest, Hungary; Doctoral School of Environmental Sciences, Eötvös Loránd University, H-1117 Budapest, Hungary; Karolinska Institutet, 171 65 Stockholm, Sweden; Uppsala University, 752 36 Uppsala, Sweden; Institute of Aquatic Ecology, Centre for Ecological Research, H-1113 Budapest, Hungary; Department of Microbiology, Eötvös Loránd University, H-1117 Budapest, Hungary; Institute of Aquatic Ecology, Centre for Ecological Research, H-1113 Budapest, Hungary; Doctoral School of Environmental Sciences, Eötvös Loránd University, H-1117 Budapest, Hungary; Department of Chemistry, Life Sciences and Environmental Sustainability, University of Parma, 43124 Parma, Italy; Institute of Aquatic Ecology, Centre for Ecological Research, H-1113 Budapest, Hungary; Swedish University of Agricultural Sciences, 750 07 Uppsala, Sweden; Uppsala University, 752 36 Uppsala, Sweden; Swedish University of Agricultural Sciences, 750 07 Uppsala, Sweden

**Keywords:** core community, droughts, interactions, keystone species, local stressors, seasonality, soda pan

## Abstract

Seasonal environmental variation is a leading driver of microbial planktonic community assembly and interactions. However, departures from usual seasonal trends are often reported. To understand the role of local stressors in modifying seasonal succession, we sampled fortnightly, throughout three seasons, five nearby shallow soda lakes exposed to identical seasonal and meteorological changes. We characterised their microeukaryotic and bacterial communities by amplicon sequencing of the 16S and 18S rRNA gene, respectively. Biological interactions were inferred by analyses of synchronous and time-shifted interaction networks, and the keystone taxa of the communities were topologically identified. The lakes showed similar succession patterns during the study period with spring being characterised by the relevance of trophic interactions and a certain level of community stability followed by a more dynamic and variable summer-autumn period. Adaptation to general seasonal changes happened through shared core microbiome of the lakes. Stochastic events such as desiccation disrupted common network attributes and introduced shifts from the prevalent seasonal trajectory. Our results demonstrated that, despite being extreme and highly variable habitats, shallow soda lakes exhibit certain similarities in the seasonality of their planktonic communities, yet local stressors such as droughts instigate deviations from prevalent trends to a greater extent for microeukaryotic than for bacterial communities.

## Teaser

Time-series study of five nearby shallow soda lakes demonstrates similar seasonal trends but microeukaryotic communities are more sensitive to local stress events than bacteria.

## Introduction

Seasonal changes of environmental parameters are the primary drivers of annual succession dynamics of planktonic communities instigating characteristic ecological processes repeated each year (Sommer et al. 1986, 2012), making seasonality the focal point of numerous aquatic microbial ecology studies (Bista et al. 2017, Lambert et al. 2018, Reji et al. 2020). Departures from usual seasonal patterns are typically explained by interannual climatic variations or long-term trends (Fuhrman et al. 2015). Seasonal changes affect shallow lakes because they have a high surface-to-volume ratio (Jeppesen et al. 2009, Cobbaert et al. 2014, Li et al. 2021). Endorheic shallow lakes are even more impacted by external environmental changes due to their intrinsic lack of outflow and consequent importance of evaporation (Frondini et al. 2019, Boros et al. 2020, Wang et al. 2022), making them ideal systems for the study of the seasonality of pelagic communities. Indications of interannual variation in the plankton dynamics of endorheic aquatic systems have been shown before (García et al. 1997, Márton et al. 2023). However, it is not known whether such variations arise because of changes of regional magnitude such as year-to-year weather and/or climate variability, or if they are the consequence of stress events of only local influence such as algae blooms or complete dry ups.

Soda lakes are a form of saline endorheic lakes characterised by carbonate, bicarbonate and sodium as dominant ions (Boros and Kolpakova 2018). These systems represent the most alkaline aquatic habitats on the surface of our planet (Jones and Grant 2000, Sorokin et al. 2011, 2014). The Kiskunság National Park (Hungary), in the central part of the Carpathian Basin (Central Europe), is a region with a particularly high density of soda pans (i.e. shallow soda lakes) (Boros et al. 2014). The climate of this region is temperate continental (Kovács and Jakab 2021), which creates considerable seasonal temperature and water level fluctuations. The drought prolonging and precipitation pattern modifying the effect of climate change (Konapala et al. 2020) is also substantial, resulting, among others, in an increasing frequency of desiccation events (Boros et al. 2013). It has been implicated that climate change-induced weather anomalies also exacerbate the interannual variation in the seasonality of the biogeochemistry of individual pans (Boros et al. 2020). Nevertheless, while the seasonal environmental changes are becoming less predictable, the close proximity of these pans means that they are still exposed to practically identical climatic and weather conditions. This makes the soda pans of Kiskunság a perfect system with which to disentangle the impact of local stress events on planktonic communities from the effect of common seasonal environmental changes.

Microbial interactions play a crucial role in microbial functioning (Tshikantwa et al. 2018) and community assembly (Leibold et al. 2022), highlighting the need to consider species interactions alongside compositional changes when studying the seasonality of aquatic microbial communities (Gilbert et al. 2011). Network-based approaches allow insight into interactions of even highly complex communities such as those of microbes (Leibold et al. 2022). Synchronous networks can assess the simultaneous occurrence and abundance of organisms, while time-shifted association networks can elucidate time-delayed impacts of species interactions (Faust et al. 2015, Fuhrman et al. 2015, Röttjers and Faust 2018). Network analysis can also identify keystone species (Fuhrman et al. 2015, Banerjee et al. 2018, Röttjers and Faust 2018), which are usually defined as hubs (i.e. highly associated species in the network) that have disproportionately high importance in the community relative to their abundance (Jordán 2009, Berry and Widder 2014, Banerjee et al. 2018).

Although microbial communities typically consist of hundreds or thousands of species (Bengtsson-Palme 2020), only a smaller fraction of the microbial species are shared among communities of a specific habitat type (e.g. soda pans) and can be defined as its core microbiome (Shu et al. 2020). The core microbiome is hypothesised to represent the functionally or ecologically most important taxa of a habitat type (Degenhardt et al. 2020, Neu et al. 2021). It has also been suggested that microbial adaptation to environmental changes through dispersal mediated species sorting also operates primarily on the core microbiome instead of the rare or sporadic (i.e. non-core) community members (Niño-García et al. 2016).

Soda lakes and pans are characterised by abundant phytoplankton (Afonina and Tashlykova 2020, Somogyi et al. 2022) and zooplankton (Burian et al. 2013, Horváth et al. 2013), which feed large amounts of migratory birds (Wagaw et al. 2019, Boros et al. 2023), while fish are typically absent (Felföldi 2020). Studies describing the planktonic microbiome of soda pans in the Carpathian Basin (Felföldi 2020, Szabó et al. 2020, 2022, Márton et al. 2023), Canada (Zorz et al. 2019), Brazil (Cotta et al. 2022), China (Zhao et al. 2020) and East Africa (Schagerl 2016) have identified unique bacterial and eukaryotic communities. The seasonal changes of the composition (Szabó et al. 2020) and function (Eiler et al. 2003) of bacterial communities of soda pans have also been described before, but the impact of seasonality on microbial interactions remains unexplored. Moreover, the seasonal succession mechanisms of bacterial and microeukaryotic communities of soda pans have never been compared. Microeukaryotes and bacteria have various inherent biological differences that might influence their seasonal dynamics, such as their different cell size and structure, generation times and life history traits. Accordingly, previous studies have demonstrated higher turnover for microeukaryotes than bacteria (Zhang et al. 2017) and that drift and dispersal play a more important role in the structuring of their communities (Logares et al. 2018, Vass et al. 2020). Meanwhile, bacterial communities tend to have higher adaptability to environmental fluctuations than eukaryotes (Liu et al. 2021).

We carried out an extensive field study evaluating the seasonal dynamics of planktonic microbial communities of five soda pans located in the same region (i.e. Kiskunság NP) throughout three seasons (spring, summer, autumn) by fortnightly sampling. We aimed to understand the impact of seasonality on the structure and interactions of bacterial and microeukaryotic communities and the contribution of core and non-core microbiome to the adaptation to environmental changes. Our primary hypothesis was that common habitat type (i.e. shallow soda lake) and identical weather and climatic exposure due to the proximity and synchronous sampling of the five sites results in analogous seasonal succession patterns, both in respect of community composition and interactions. We further hypothesised that adaptation to seasonal changes happens primarily through species recruitment from the core rather than the non-core community, while non-core taxa are to a large extent involved in the response to local stressors. Finally, we hypothesise that bacterial communities are less affected by local stress events than eukaryotic communities.

## Materials and Methods

### Sample collection and environmental parameters

**Figure 1.**
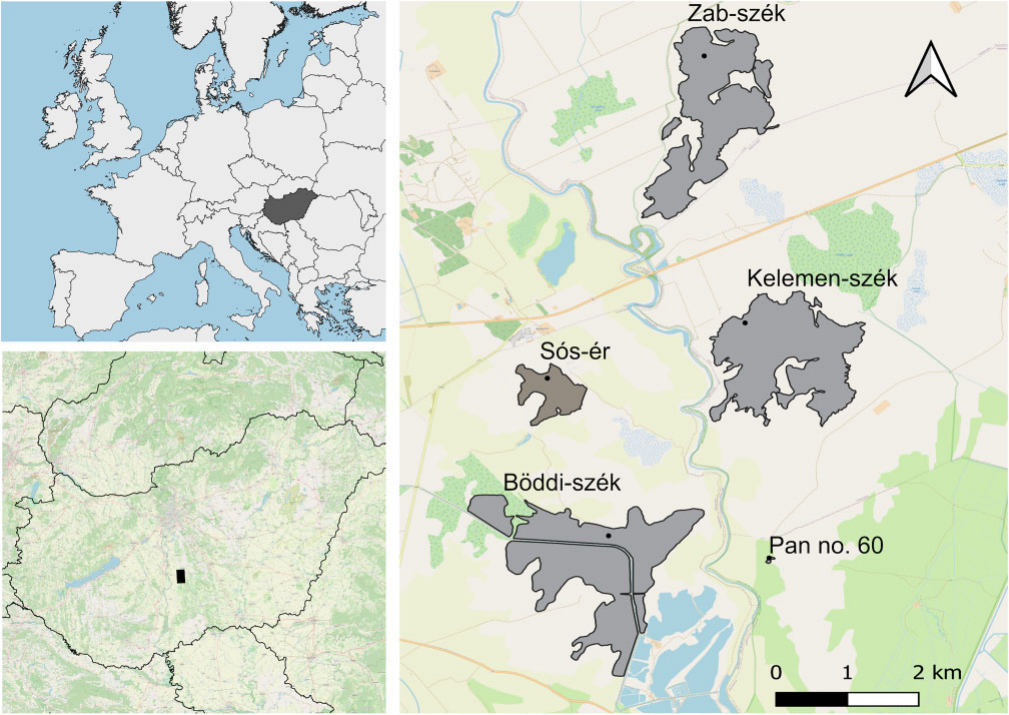
Location of the sampling sites in Kiskunság National Park, Hungary: Böddi-szék (46°46.07′ N, 19°09.007′ E), Kelemen-szék (46°47.893′ N, 19°10.440′ E), Sós-ér (46°47.341′ N, 19°8.679′ E), Zab-szék (46°50.190′ N, 19°10.283′ E) and an unnamed pan (Pan no. 60, 46°45.818′ N, 19°10.828′ E). (The map was created with QGIS Geographic Information System (v. 3.28.1), QGIS Development Team (2022). Open Source Geospatial Foundation Project. http://qgis.osgeo.org).

We selected five soda pans located within an area of 14 km^2^ of Kiskunság National Park, Hungary: Böddi-szék, Kelemen-szék, Sós-ér, Zab-szék and an unnamed pan (Pan no. 60) (Fig. 1). Two different subtypes of soda pan are distinguished in this region: the prevailing turbid-white subtype (referred to as turbid from here on), which is characterised by high amounts of suspended inorganic clay particles, and the less common non-turbid, brown subtype (referred to here as brown), which has a characteristic brown colour due to high organic material content and a low amount of suspended inorganic material (Boros et al. 2014). Among the sampled pans Sós-ér was brown, while the other pans were turbid.

**Figure 2.**
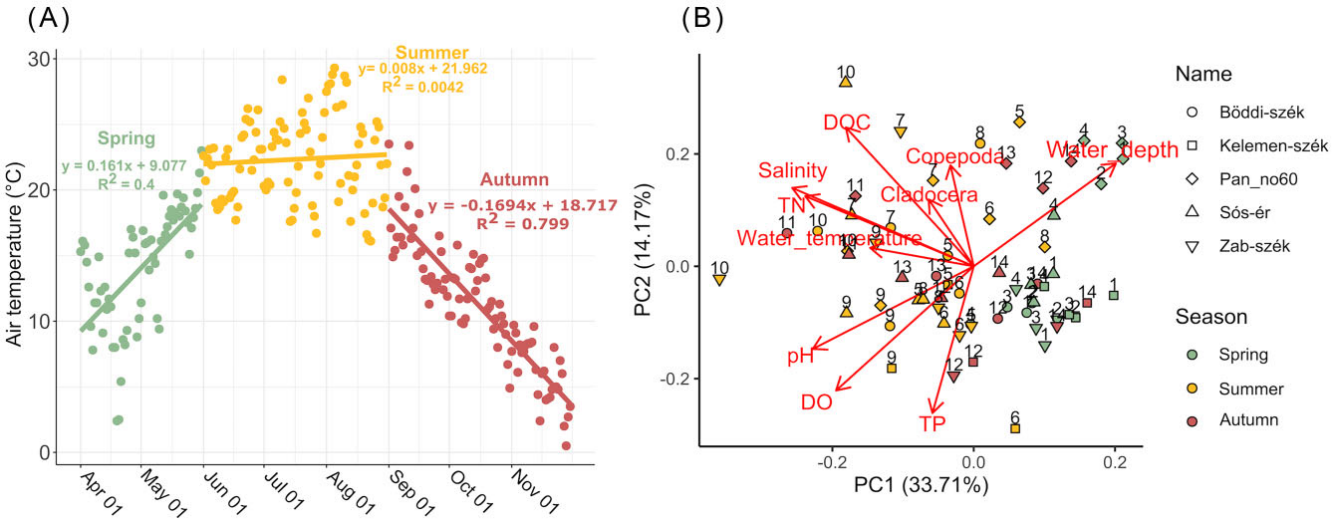
Seasonal changes of environmental parameters. **(A)** Air temperature trend during the sampling period based on the daily air temperature measurements of the nearby Soltszentimre meteorological station; **(B)** PCA biplot of the environmental variables measured in the soda pan samples. Numbers represent the sampling times, different symbol shapes the five pans, while different colours represent the three studied seasons (green for spring, yellow for summer and red for autumn).

The pans were sampled fortnightly through three seasons: spring (sampling time 1–4), summer (sampling time 5–10) and autumn (sampling time 11–14) from 12 April to 14 November in 2017 to cover the main productivity period of the year and to ensure ice-free conditions (Fig. 2A). All samples were pooled by proportionally mixing water collected from 1 cm below the surface, from at least five different points near the deepest part of the pans. For microbial community analyses, water samples were collected into a 1-litre sterile bottle after filtering through a 40 µm mesh size plankton net to remove large zooplankton. Larger algae are practically absent from these pans; the mean contribution of pico-sized (cell diameter <2 µm) algae to total phytoplankton biomass is ∼85% in the turbid pans (Somogyi et al. 2022), while in the brown soda pans nanoplanktonic (2-20 µm) algae have a similar contribution (∼80%), with a moderate amount of picophytoplankton (∼8%) (Szabó et al. 2020, Somogyi et al. 2022). The water samples were transferred to the laboratory in a cooling box, where planktonic microbial cells were collected onto 0.1 µm pore size filters (MF-Millipore membrane filter) by filtering 30 ml water for turbid pans and 50 ml for the brown pan. The filters were stored at –80°C until further processing.

Temperature, pH (SenTix 41 electrode), conductivity (TetraCon 325 cell) and dissolved O_2_ (CellOx-325 electrode) were measured on site with a MultiLine Handheld Meter model 340i (WTW, Weilheim in Oberbayern, Germany). Meteorological data from the Soltszentimre station (within 15 km distance from all sampling sites) were provided by the Hungarian Meteorological Service. Further environmental parameters were determined in the laboratory. Chlorophyll a was measured based on a previously published study (Mentes et al. 2018), while total phosphorus (TP), total nitrogen (TN) and dissolved organic carbon (DOC) concentrations were determined as described in Nydahl et al. (2019). Electrical conductivity data (EC) were converted to salinity based on a previously published equation estimated specifically for soda lakes of the same region (Total ions (g/L) = 0.792 * EC (mS/cm) + 179) (Boros et al. 2014). A total of 20 litres of water collected at different points around the deepest part of the open water area was sieved through a 40 µm mesh size plankton net and preserved in 70% ethanol until the abundance and composition of zooplankton was determined (Horváth et al. 2014). Subsampling was used to reduce the counting effort in the case of samples containing more than 300 specimens, otherwise all the specimens were counted. The abundance of cladocera and copepoda was calculated as individuals per litre.

### Community analysis

DNA was extracted using DNeasy PowerSoil Kit (QIAGEN) according to the manufacturer's instructions. Microeukaryotic and bacterial community composition was determined by amplification and sequencing of the V4-V5 region of the 18S rRNA gene and V3-V4 region of the 16S rRNA gene, respectively. No archaea-specific PCRs were carried out, because they represent only a minor fraction of the prokaryotic community in these sites (Korponai et al. 2019, Szabó et al. 2020). Amplification and library preparation for amplicon sequencing were carried out based on previously published studies (Székely et al. 2019, Vass et al. 2020) (Text S1.), while sequencing was performed at the Swedish National Genomics Infrastructure (Uppsala, Sweden) on Illumina MiSeq platform (Illumina Inc, San Diego, CA, USA) in a 2 × 300 bp paired-end format and with v3 chemistry.

Sequence read processing, alignment and taxonomic assignments were carried out using mothur v. 1.41.1 (Schloss et al. 2009). Operational taxonomic units (OTUs) were assigned at 99% similarity cutoff and rarefied OTU sets were created as a basis for subsequent analyses. For the amplified region and based on previous studies of the surveyed habitat, this cutoff value was found to be suitable to avoid most diversity estimation biases caused by clustering different species to the same OTU or splitting organisms having multiple rRNA copies to separate clusters (Johnson et al. 2019, Schloss 2021). The seventh sampling time of Pan no. 60 was discarded from the 18S rRNA gene amplicon dataset due to low sequencing quality. Reads were subsampled to the read number of the samples with the lowest sequence counts (62 samples in the 18S rRNA amplicon set, n = 2407; and 63 samples in the 16S rRNA amplicon set, n = 3188). A detailed description of the sequence analysis is provided in Supplementary Text S2.

OTUs present in all five studied soda pans were defined as core5 and OTUs shared between the four turbid soda pans as core4. OTUs not shared between the pans were defined as non-core5 and non-core4, respectively.

### Statistical analyses

The measured environmental variables were scaled to unit variance and compared using principal component analysis (PCA). The communities of the turbid and brown pans were compared by one-way permutational multivariate analysis of variance (PERMANOVA, ‘adonis’ function, permutations = 999) based on Bray-Curtis (BC) dissimilarity of the microeukaryotic OTUs (eOTUs) and bacterial OTUs (bOTUs), while differences in community compositions among seasons and pan identity were tested by two-way PERMANOVA (permutations = 999). Non-metric multidimensional scaling (NMDS) based on BC distance was used to visualise the microeukaryotic and bacterial communities, while the ‘envfit’ function was applied to plot significantly fitted (*P* < 0.05) environmental vectors onto the NMDS ordinations. Mantel-tests were implemented to verify the results of ‘envfit’ by identifying significant correlation between microeukaryotic, and bacterial communities and environmental variables, and zooplankton abundance. Highly collinear variables were identified based on Pearson correlation (|r| > 0.7) (Dormann et al. 2013). To assess the structural temporal dynamics of each pan, BC dissimilarity between sampling occasions (i.e. time distance decay curve) was calculated for both 18S and 16S rRNA gene OTUs (i.e. eOTUs and bOTUs, respectively) and the BC dissimilarity between consecutive sampling occasions was considered as a proxy of community turnover. The effect of drought on community turnover of microeukaryotes and bacteria was tested by comparing the BC dissimilarities between consecutive sampling times during the drought period in late summer and autumn (i.e. between sampling times 7 and 14) for the three non-drying pans (Böddi-szék, Pan no. 60 and Sós-ér) and the two drying pans that underwent various drought events during this period (Zab-szék and Kelemen-szék). Analysis of variance (ANOVA) with Tukey's post-hoc test was used to test the differences of environmental variables, core OTU contribution and turnover between soda pans and seasons.

All statistical analyses were carried out by R (v. 4.2.2) (R Core Team 2022) using the ‘vegan’ package (v. 2.6.4) (Oksanen 2017) for multivariate and ‘tidyverse’ (v. 1.3.2) for univariate analyses (Wickham et al. 2019).

### Network analyses

The extended Local Similarity Analysis (eLSA) is a robust time series analysis tool that not only detects microbial associations that are present throughout the study period (i.e. global correlations), but also those that only occur in a subinterval of the time series (i.e. local associations). Furthermore, eLSA considers both associations between taxa that coexist in time (i.e. co-occurence) and time-shifted or time-lagged correlations (Ruan et al. 2006, Xia et al. 2011, Fuhrman et al. 2015). To better understand synchronous and asynchronous interactions, we generated two eLSA networks of the microeukaryotic and bacterial communities of each soda pan: one using the synchronous correlations (i.e. co-occurrence networks, delay 0) and another using only time-shifted correlations (delay 1 or -1). The eLSA (v. 1.02) was carried out for each pan using the default settings, except for adjusting the delay limit to 1 and data normalisation with the percentileZ function. To reduce the complexity, only OTUs with >1% relative abundance in at least one sample and present with more than 10 reads in at least three different subsampled samples were included in the network analysis. For both global (Spearman's rank correlation coefficients [SSCC]) and local associations (local similarity scores [LS]), only strongly significant (*P* < 0.01 and q < 0.01) correlations were included. Network visualisation was carried out with Cytoscape v. 3.8.2 using the edge-weighted, spring-embedded layout (Shannon et al. 2003).

To identify OTUs in important network positions and to quantify their centrality, we used the weighted topological importance (WI) measure generalised by Jordán et al. (2006). This index calculates the number of neighbours and the number of their neighbours, in an additive and multiplicative way, while considering the strength of the interactions. To differentiate keystone OTUs (i.e. those OTUs that play a key role in the network and their removal would drastically impact the structure of the network), we considered indirect interactions up to three steps (WI^3^) and selected OTUs with WI_i_^3^ > 1 (Jordán et al. 2006, Berry and Widder 2014). If less than six OTUs fulfilled this requirement in a network, the selection was expanded to include OTUs with WI_i_^3^ ≥ 1. In the network of interactions, one can speak of ‘negative keystone OTUs’ that are richly connected via negative associations and, contrariwise, ‘positive keystone OTUs’ that are rich in positive associations with others. We note that positive associations can be strongly transitive (AB and BC frequently implies AC), while negative associations are rarely transitive (AB and BC generates a positive AC association instead of AC in the negative network). Two heatmaps were generated using the 'ComplexHeatmap' R package (v. 2.12.1) (Gu et al. 2016) to visualise the z-score transformed abundance of the clustered keystone OTUs of each pan with two columns for annotation of the overall direction of their interactions and their preferred season determined as the season when their mean z-score transformed abundance was positive.

## Results

### Environmental parameters

Meteorological data revealed typical seasonal air temperature dynamics with an increasing trend in spring (rate: +0.16°C/day, mean: 14.1°C), no trend in summer (mean: 22.3°C) and a decreasing trend in autumn (rate: -0.17°C/day, mean: 11.3°C) (Fig. 2A). The measured environmental parameters had values and followed trends previously described for the soda pans of this region (Table S1) (Boros et al. 2014, 2020, Felföldi 2020, Szabó et al. 2020). In general, for samples collected in spring, similar environmental parameters have been measured, while in summer and autumn variation increased both between sampling times and sites (Fig. 2B, Figure S1). Water depth varied greatly (1.5–46.0 cm) during the sampling period with the deepest water levels measured during spring. Moreover, Zab-szék and Kelemen-szék were completely dry on some occasions (sampling time 7, 8, 10, 11 and 13 for Kelemen-szék; and 11 and 13 for Zab-szék), making water sampling impossible. According to the PCA biplot, water depth was negatively related to the concentration of soluble compounds, pH, as well as copepoda and cladocera abundance, which had the highest values in mid-summer, while pH, DO and TP were elevated in summer and early autumn (Fig. 2B, Figure S1). Despite the common trends, only salinity and TN, and salinity and DOC, were highly collinear. Although the PCA did not markedly distinguish the environmental parameters of the brown from the turbid pans (Fig. 2B), Sós-ér had, on average, significantly deeper waters and higher TN concentrations as well as the highest median DOC, and lowest TP and pH (Figure S1). For a detailed description of the trends in environmental parameters, check Text S3.

### Community composition

**Figure 3.**
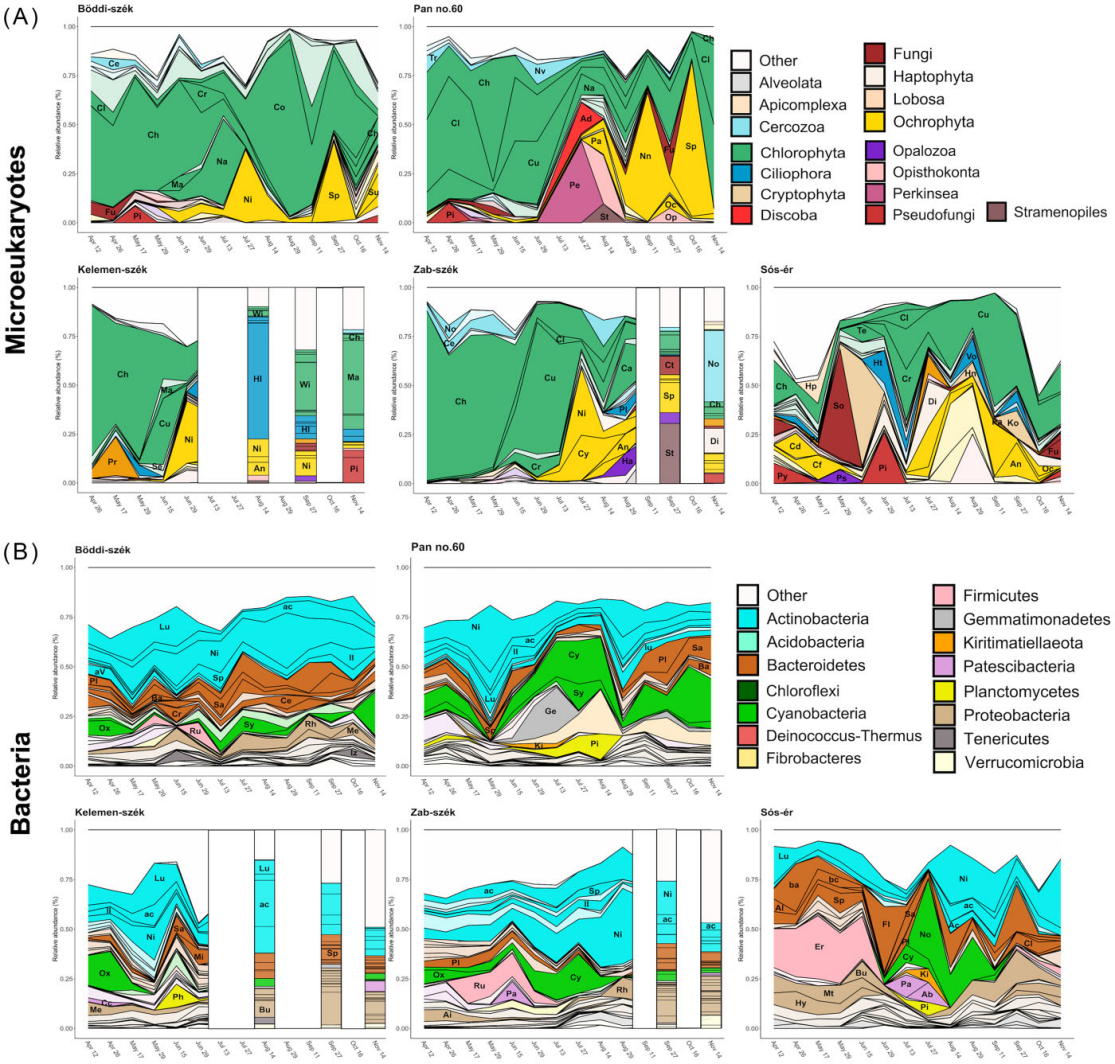
Microbial community dynamics of the **(A)** microeukaryotic and **(B)** bacterial OTUs with >1% relative abundance in at least one sample of the given pan coloured according to the corresponding phyla. OTUs with > 5% relative abundance in at least one sample are highlighted and indicated by the abbreviated name of the corresponding microeukaryotic genera or bacterial clade, respectively. A key to the abbreviations can be found in Supplementary Table S2.

A total of 4524 microeueukaryotic OTUs (eOTUs) and 4241 bacterial OTUs (bOTUs) were identified from the sequencing data. In all five pans the three most abundant microeukaryotic phyla were Chlorophyta (mean relative abundance: 53%; range: 8%–98%), Ochrophyta (15%; 0%–82%) and Fungi (4%; 0%–59%). Within the Chlorophyta phylum the single most abundant eOTU in all pans was a green alga affiliated to the *Choricystis* genus (abbreviated name Ch, 21%; 0.1%–86%). This *Choricystis* eOTU showed clear seasonality with a mean abundance in spring of 46%, which decreased to 9% in summer and only 3% in autumn, although by November it increased again to 7% (Fig. 3A). Actinobacteria (30%; 7%–69%) and Cyanobacteria (10%; 0%–47%) were the most abundant bacterial phyla in all pans. Within Actinobacteria, the most abundant bOTU (Ni, 4%; 0%–30%) belonged to the Nitriliruptoraceae family. Meanwhile, OTUs identified as Cyanobium_PCC-6307 (Cy) and *Synechococcus*_MBIC10613 (Sy) were the most frequent cyanobacterial lineages (Fig. 3B).

The seasonal community dynamics of the brown Sós-ér differed from those of the turbid pans. Here, in spring, a bOTU belonging to the family Erysipelotrichaceae (Er) was dominant, while in the second half of summer there was a cyanobacterial bloom by a filamentous nitrogen-fixing *Nodularia*_PCC-9350 (No) bOTU. Some eOTUs, such as the members of Chrysophyceae Clades D (Cd) and F (Cf) or the parasitic fungus genus *Pythium* (Py), were also only dominant (> 1%) in Sós-ér (Fig. 3). Meanwhile, the turbid pans had relatively similar community dynamics; the only exception were the drastic shifts in the microeukaryotic community composition observed following the desiccation-refillment events in the drying pans (i.e. Kelemen-szék and Zab-szék) when specific eOTUs became dominant (e.g. after the first desiccation in Kelemen-szék the ciliate *Halteria* [Hi] had 59% abundance, while in Zab-szék a Stramenopiles [St; 31%] eOTU became the most abundant after the first and a novel clade of Rhizaria [No; 33%] after the second drought).

### Core microbial community

**Figure 4.**
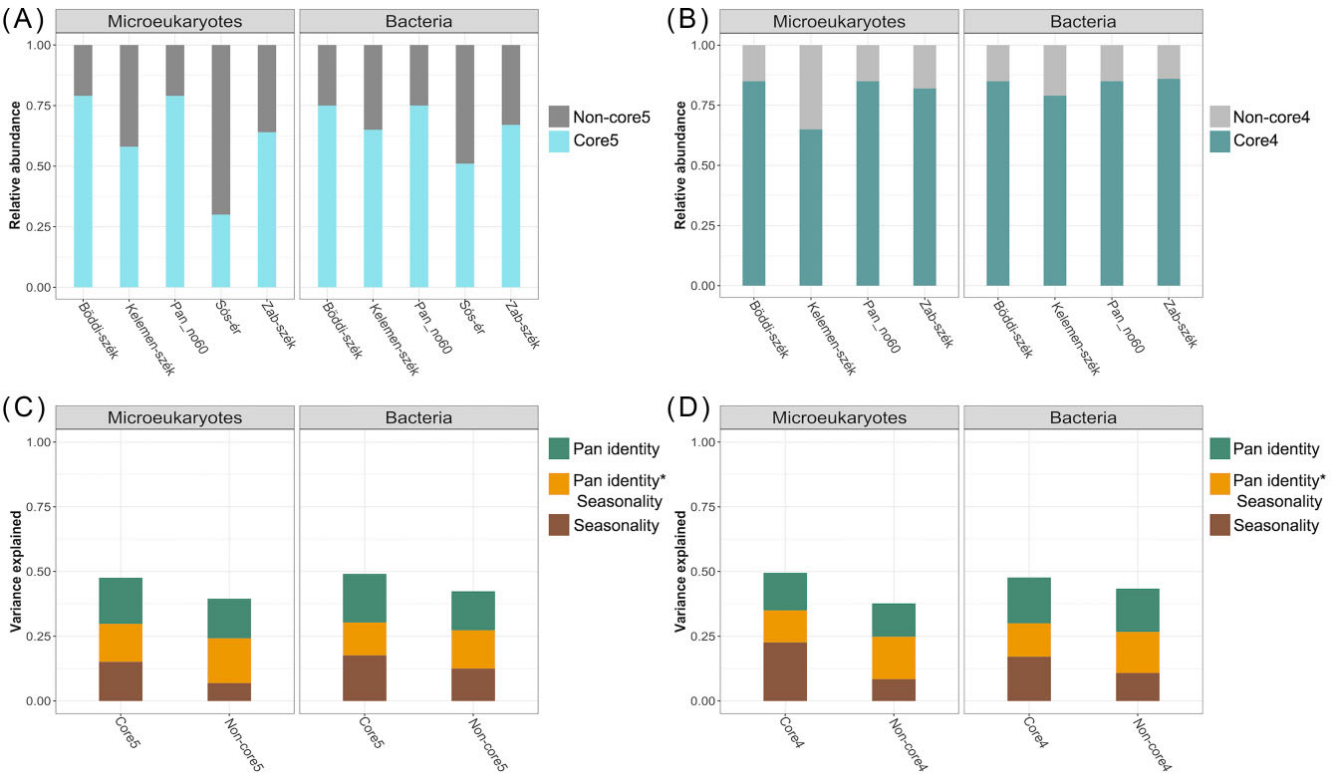
Relative abundance of the microeukaryotic and bacterial core communities in each pan. **(A)** OTUs shared by all pans (core5) and **(B)** OTUs shared by the turbid pans (core4). Variance of community structure explained by pan identity, seasonality and the interaction of the two factors (i.e., Pan identity* Seasonality) in the case of **(C)** core5 and **(D)** core4 communities.

From the almost 9000 identified OTUs, only 97 eOTUs and 191 bOTUs were detected in all five pans; however, these core5 OTUs represented 62% and 67% of the 18S and 16S rRNA gene reads, respectively (Figure S2). The core5 OTUs were predominant in turbid pans but not in Sós-ér, where core5 eOTUs and bOTUs constituted only 30% and 51% relative abundance, respectively (Fig. 4A). When considering all the time points, the contribution of core5 eOTUs to the communities of Sós-ér was substantially lower than to the turbid pans (*P* < 0.001), while, despite the lower contribution of core5 bOTUs to Sós-ér during spring, there was no significant difference between the brown and the turbid pans regarding the relative abundance of core5 bOTUs (*P* > 0.05).

The core4 OTUs shared only by the turbid pans consisted of 5–10 times more OTUs than core5 (952 eOTUs and 988 bOTUs) and represented 80% and 84% of the respective reads (Fig. 4B). Differences among the turbid pans in respect of core OTUs were also detected. More precisely, the relative abundance of non-core OTUs was higher in the drying pans (Kelemen-szék and Zab-szék) than in the non-drying pans (Böddi-szék and Pan no. 60), and this difference was more notable for eOTUs than for bOTUs (Fig. 4A and B). Furthermore, the contribution of both core5 and core4 eOTU reads went markedly down after drought events, while core5 and core4 bOTUs showed a relatively stable contribution over time, irrespective of desiccation (Figure S3).

### Drivers of community changes

**Figure 5.**
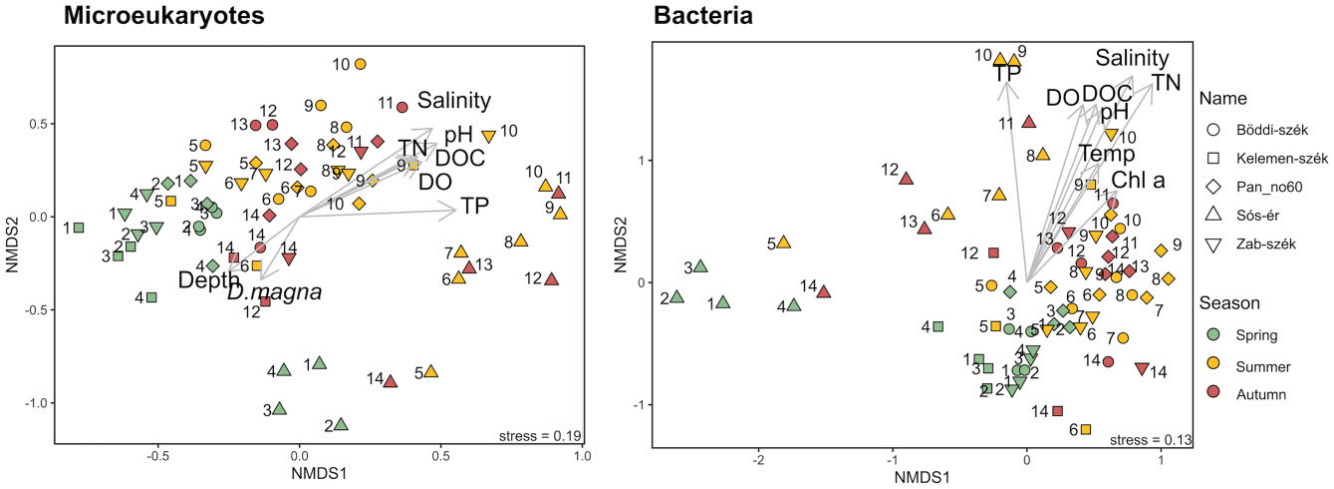
Non-metric multidimensional scaling (NMDS) ordination of the microeukaryotic and bacterial planktonic communities of the five soda pans based on Bray-Curtis distance with the significantly fitted environmental parameters.

**Figure 6.**
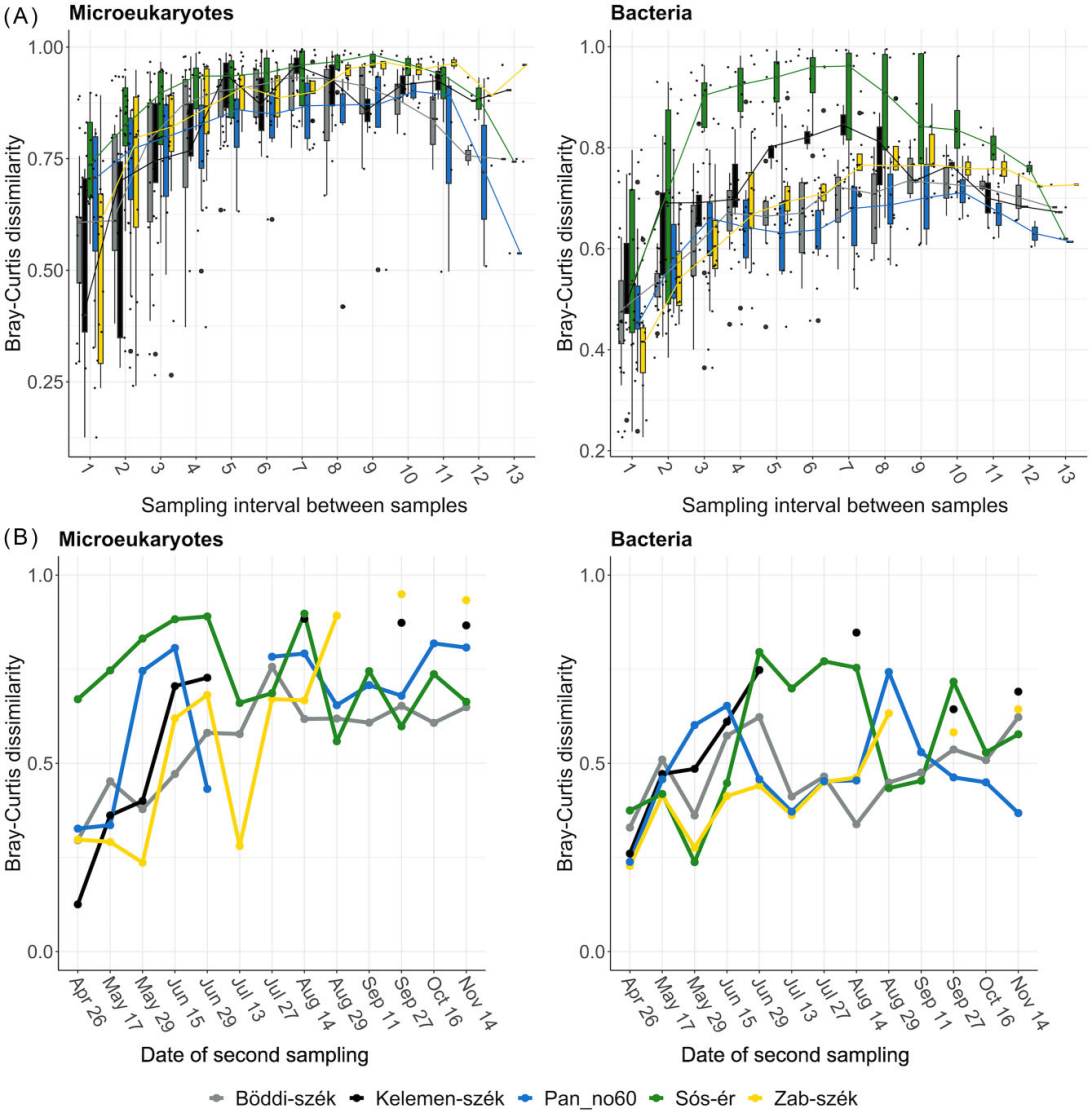
**(A)** Bray-Curtis (BC) dissimilarity index between consecutive samplings as proxy for community turnover. The x-axis indicates the date of the latter sampling date within consecutive sampling pairs. **(B)** Time distance decay curve of community dissimilarity during the sampling period. Boxplots represent the pairwise BC dissimilarity index between samples of the same pan taken at the given sampling interval. The x-axis shows the sampling interval between sampling events.

The ‘envfit’ analysis significantly (*P* < 0.05) fitted salinity, pH, DOC, TN, TP and DO on the NMDS plots of both the microeukaryotic and bacterial communities. However, water depth and *Daphnia magna* abundance were significantly fitted only on the microeukaryotic NMDS, while water temperature and chlorophyll were only significant for bacterial communities (Fig. 4). The importance of DOC, TN and TP for both microeukaryotic and bacterial communities, and water temperature for bOTUs, was enforced by significant Mantel tests, although water temperature and DOC for bOTUs were only marginally significant (*P* = 0.046). Meanwhile, salinity and DO were only significant for eOTUs (Table S3). The season of sampling had a significant effect on the communities (PERMANOVA Microeukaryotes: R^2^ = 0.144, *P* = 0.001; Bacteria: R^2^ = 0.115, *P* = 0.001) and the differences between seasons were also significant, with the strongest differentiation of spring communities from summer and autumn (Table S4). Interestingly, the last autumn samples (sampling 14) appear close to the spring samples on both microeukaryotic and bacterial NMDS plots, suggesting similarity between the spring and late autumn samples (Fig. 5). This was also supported by the time distance decay curve, which decreased again for the sample pairs with a high time difference in almost all cases, except for the microeukaryotic communities of the two drying pans (Fig. 6A). The NMDS plots and the PERMANOVA analysis testing the impact of soda pan subtype (i.e. brown or turbid), clearly separated the communities of the brown Sós-ér from those of the turbid pans (Microeukaryotes: R^2^ = 0.086, *P* = 0.001; Bacteria: R^2^ = 0.145, *P* = 0.001).

For core communities, the two-way PERMANOVAs assessing the effect of pan identity and sampling season, irrespective of the analysed domain (microeukaryotes or bacteria) and the number of lakes included (all five or only four turbid), always explained more variance than for the non-core communities (Fig. 4C and D, Table S5). Furthermore, seasonality explained more variance for core communities than for non-core communities, particularly in the case of microeukaryotes (Fig. 4C and D, Table S5).

### Community turnover

Both seasonality and lake identity had a significant effect on turnover (i.e. BC dissimilarity between sampling times) (Table S6). Spring was characterised by significantly lower values than summer and autumn, indicating a period of stability in spring (Fig. 6B). More precisely, while in Sós-ér the microeukaryotic turnover was high throughout the study period, in the turbid pans in spring the microeukaryotic BC dissimilarity between sampling times was mostly low (< 0.5). In late spring and early summer, eOTU turnover substantially increased for Pan no. 60, Zab-szék and Kelemen-szék and gradually for Böddi-szék, indicating major shifts in the microeukaryotic community structure of the turbid pans between the two seasons. While in June and July there were single consecutive sampling times with low BC dissimilarity in Zab-szék and Pan no. 60, starting from mid-July to the end of the study period, microeukaryotic turnover remained high (> 0.5) in all pans. In the case of the drying pans (i.e. Zab-szék and Kelemen-szék), the eOTU BC dissimilarity was even higher, indicating that the microeukaryotic community composition after the refillment was drastically different from the communities before the drought, which was further corroborated by the significant difference (*P* = 0.001) in the microeukaryotic turnover in this period between the non-drying and drying soda pans.

The bacterial BC dissimilarity between consecutive samplings was overall lower (mean 0.5) than for eOTUs (mean 0.6) (Fig. 6B). Bacterial turnover increase between spring and summer occurred only for the brown Sós-ér, and Kelemen-szék, the turbid pan with the most desiccation events. After this, Sós-ér maintained high bacterial turnover until mid-August, while the turnover of the turbid pans remained relatively low throughout the summer. In the drought period of late summer and autumn, opposite to the microeukaryotic communities, bacterial turnover of the drying pans was not higher (*P* = 0.162) than those of the non-drying pans. The only desiccation-refillment event with notable bacterial turnover increase was the first drought of Kelemen-szék.

### Microbial interactions

**Figure 7.**
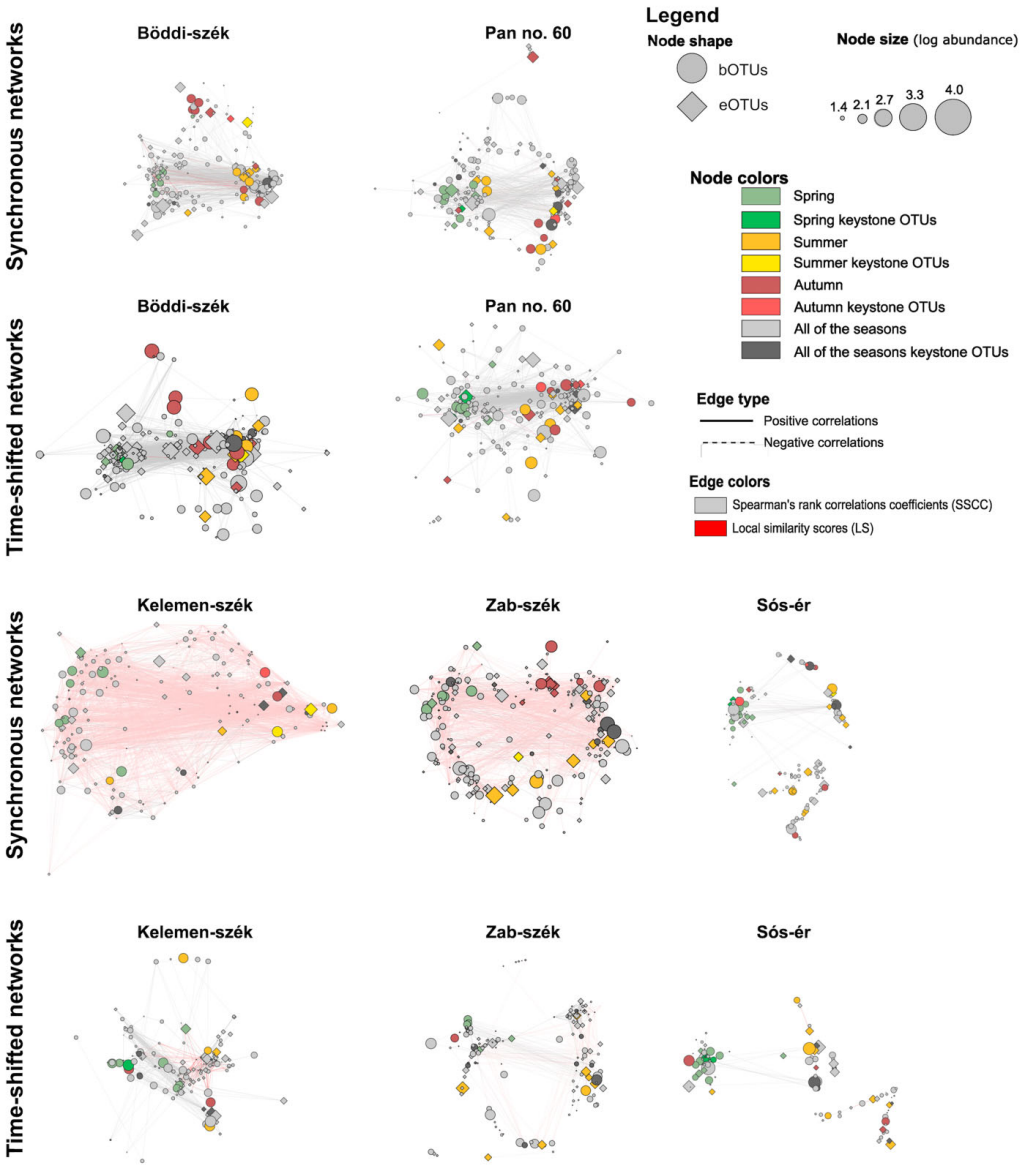
Synchronous and time-shifted networks of bacterial and microeukaryotic OTUs of the soda pans. The colouring of the network nodes was based on the preferred season of the OTUs (i.e. the season when the OTU was substantially more abundant than in the rest of the sampling period). The preferred season of each OTU was defined as the season when the difference between their mean relative abundance in the given season and their mean relative abundance in the entire sampling period was larger than the standard deviation of their relative abundance in the entire sampling period ((MEAN_season_—MEAN_study period_) > SD_study period_). Green indicates spring as preferred season, yellow summer and red autumn. Grey denoted OTUs that lacked a distinct preferred season. The keystone OTUs are distinguished by brighter colours. The colouring of the edges is based on the correlation type (grey edges = SSCC, pink edges = LS), while line type of the correlations corresponds to their direction (solid edges = positive correlation, dashed edges = negative correlations).

All networks had more positive correlations than negative, irrespective of the pan or type of network (Table S7). The networks of the non-drying turbid pans (i.e. Böddi-szék and Pan no. 60) had various properties not shared with the networks of the other pans: (1) both their synchronous and time-shifted networks had two distinct clusters of hubs densely connected with mostly SSCC edges (i.e. global correlations) (Fig. 7), and (2) their synchronous and time-shifted networks had a similar number of nodes, but the time-shifted had more edges and higher density (Table S7).

Meanwhile, the networks of the two drying turbid pans (i.e. Kelemen-szék and Zab-szék) also shared various similarities, partly in contrast to the networks of the other pans: (1) their synchronous networks had more edges and nodes, and were denser than their time-shifted networks; (2) the nodes of their synchronous networks were densely connected with mostly LS edges (i.e. local correlations); and (3) the topology of their time-shifted networks, especially in the case of Zab-szék, was more fragmented than their synchronous networks (Fig. 7, Table S7).

The networks of the brown Sós-ér shared properties with the non-drying turbid pans such as the similarity of the synchronous and time-shifted network, and higher density of the time-shifted network. They also had similarities to the networks of the drying turbid pans, such as the higher number of nodes and edges in the synchronous networks. However, the networks of Sós-ér were also distinguished from all turbid networks because they had the lowest numbers of edges, nodes and neighbours, and overall low density (Table S7). The topology of both of the networks of Sós-ér was highly fragmented, including an additional hub corresponding to the *Nodularia* bloom period (Fig. 7).

**Figure 8.**
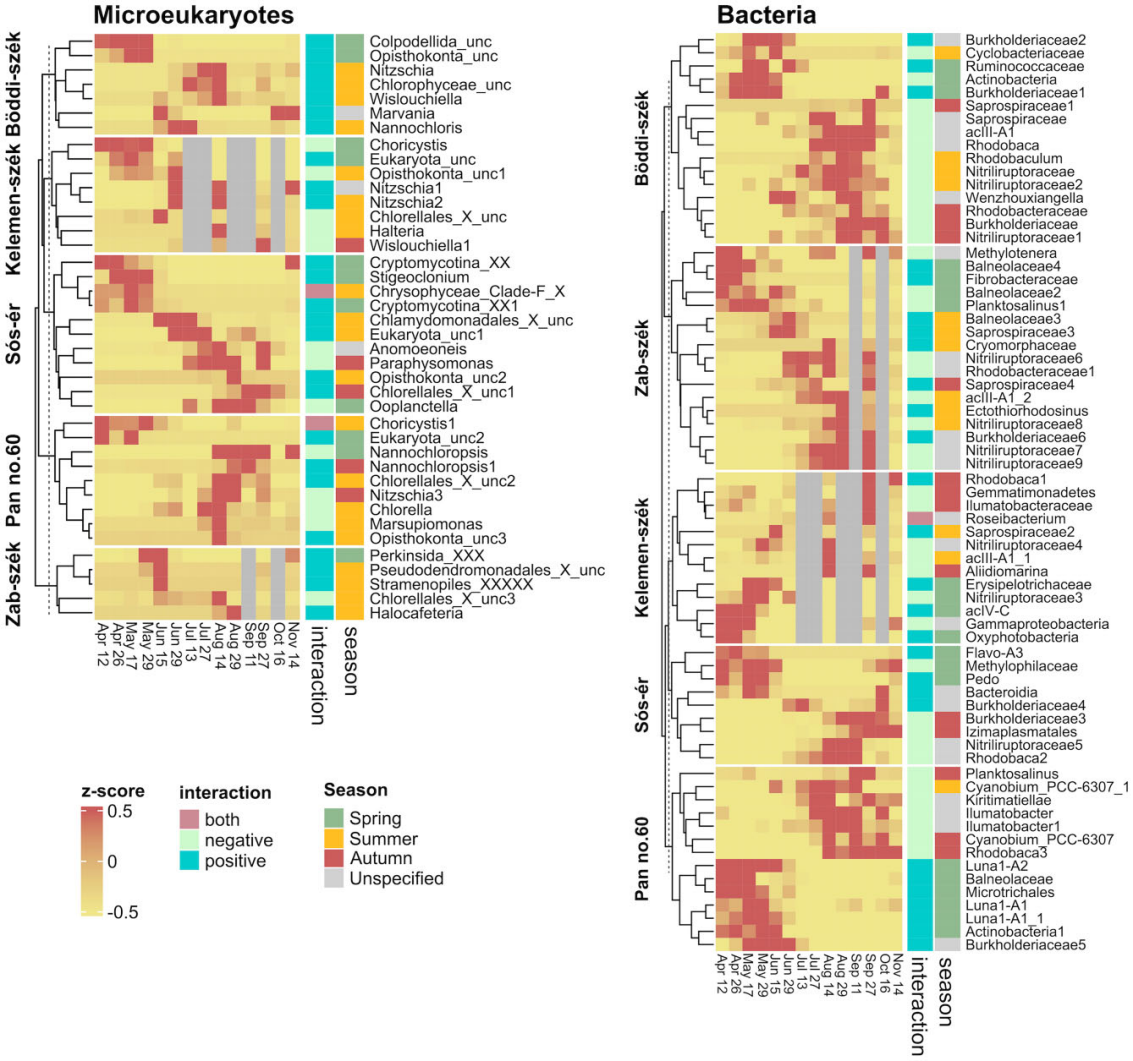
Clustered heatmap of the z-score transformed abundance of the microeukaryotic and bacterial keystone OTUs of the networks of each pan annotated by the overall direction of their interactions. Season represents the preferred season of the keystone OTU (i.e. the season when the OTU was substantially more abundant than in the rest of the sampling period). The preferred season was determined as the season when the mean z-score transformed abundance of the OTU was positive. If more than one season had positive mean z-score transformed abundance, no preferred season was specified (unspecified).

Although microeukaryotic and bacterial OTUs were both identified as keystone OTUs in all networks, the majority of keystone OTUs were bacteria and there were more bacterial keystone OTUs in the turbid pans than in Sós-ér (Fig. 8). Only one-third (36%) of eukaryotic keystones was core5, while for the turbid pans 58% were core4. Among the keystone bOTUs, the majority were core5 (79%) and almost all were core4 (91%) in the turbid pans. Many keystone OTUs were assigned to phyla with high relative abundance such as Chlorophyta, Ochrophyta and Fungi for keystone eOTUs and Actinobacteria for bOTUs. However, Cyanobacteria were underrepresented among keystones with even very abundant taxa such as *Nodularia*_PCC-9350 and *Synechococcus*_MBIC10613 was not identified as a keystone. Furthermore, the highly abundant Erysipelotrichaceae OTU in Sós-ér was also not a keystone. Meanwhile, among the less abundant (<0.1% of reads) keystones, there were two eOTUs assigned to the parasitic Cryptomycotina order.

There were various differences among positive and negative keystone OTUs (Fig. 8). First, positive keystones were more common among those with highest abundance in spring (74% of spring keystones), in summer the number of positives and negatives was similar, while among those abundant in autumn, negatives were more common (80%). For bOTUs, taxonomic differences between positives and negatives were also noted. On the phylum level, Bacteroidetes (10) was the most common among positive keystones, followed by Proteobacteria (seven) and then Actinobacteria (six), while for the negative OTUs Actinobacteria was the most common (17), followed by Proteobacteria (13) and then Bacteroidetes (six). Differences were obvious also at lower taxonomic levels as negative actinobacterial keystones were present in high abundance throughout the study period and primarily belonged to the Nitriliruptoraceae (10) and acIII-A1 (three) lineages, while the positives were only abundant in spring and belonged to Luna1-A (three) and acIV-C (one). Similarly, the most common family for negative proteobacterial keystones was summer and autumn abundant Rhodobacteraceae, while for positives it was Burkholderiaceae (five) in all three seasons.

No substantial taxonomic difference was notable between keystone eOTUs of the synchronous and time-shifted networks. However, for bacterial keystones, Actinobacteria were more common in the synchronous networks than in the time-shifted ones (16 vs. 8), while Bacteroidetes keystones were more common in time-shifted networks (10 vs. 6).

## Discussion

Planktonic microbial communities of five shallow soda lakes exposed to identical climatic and meteorological conditions were evaluated by a synchronous time series analysis. The results demonstrated that the pans shared a core microbial community and similar seasonal dynamics, both in respect of community composition and interactions. However, the extent of shared microbiome and trends was not uniform across the pans. Substantial differences were identified based on habitat subtype (i.e. brown or turbid). Common seasonal succession trajectories were also disrupted by local stressors (e.g. desiccation). Such events prompted a stronger response from microeukaryotic than from bacterial communities.

### Differences of the brown soda pan

Although both the brown and turbid pans investigated in this study represent characteristic soda pan habitats (i.e. high pH, specific ion composition and shallowness) (Boros et al. 2014), the brown Sós-ér is also characterised by extremely high coloured DOC matter and DOC concentrations, and abundant shoreline vegetation (Boros et al. 2020). Furthermore, it has been previously demonstrated that Sós-ér harbours markedly different microbial communities than the turbid Zab-szék (Szabó et al. 2017, 2020). In our study, Sós-ér also had significantly different microeukaryotic and bacterial communities as well as significantly deeper waters, and higher TN concentrations combined with low TP and pH and high DOC levels. Our analyses (i.e. envfit and Mantel tests) suggested that the primary drivers of the distinctive microbial communities of the brown pan in this period were the different nutrient concentrations (i.e. TP, TN, DOC). Sós-ér also had a substantially lower number of shared OTUs (core5) than the turbid pans. The contribution of core5 eOTUs to the Sós-ér community was low throughout the study period and various unique microeukaryotic taxa were detected with high relative abundance only in this pan, indicating that different soda pan subtypes exert strong selective forces on microeukaryotes. Meanwhile, from mid-summer, the contribution of core5 bOTUs to the Sós-ér community was similarly high as in the turbid pans, suggesting for bacteria more similar selection processes and no dispersal limitation between the five pans. Sós-ér also had various strikingly different intrinsic characteristics such as fragmented, low density and connectivity networks, as well as a high turnover of eOTUs that indicate highly dynamic communities. Interestingly, the bOTUs of Sós-ér had a high turnover only during summer and autumn, while in spring the bacterial turnover was low, together with the low core5 bOTU contribution in this period, suggesting a distinctive but stable spring bacterial community. This spring community was characterised by the high abundance of *Erysipelotrichaceae* UCG-004 bOTU, a taxon that was previously reported from soda pans of this region (Szabó et al. 2020), but has been otherwise described primarily from the digestive systems of mammals and insects (Tegtmeier et al. 2016, Cox et al. 2017, Wu et al. 2021).

### Common seasonal trends

Seasonality significantly affected the microeukaryotic and bacterial communities of all five pans and all studied seasons had distinctive communities. Furthermore, despite the differences of Sós-ér, various common attributes of seasonal microbial succession could be identified. First, a circular trajectory of seasonality indicated by the resemblance of early spring and late autumn communities was detected for all pans, which was driven by low water temperature and deeper waters and consequently low pH and low concentration of solutes. The seasonal variation explained by the core vs. non-core communities also showed similarities, irrespective of including Sós-ér in the analyses (i.e. core5 or core4). More specifically, the higher seasonal variance explained by the core than by the respective non-core communities supported our hypothesis in respect of seasonal adaptation happening primarily through species recruitment from the core community.

Further similarities of the seasonality of the five pans were the analogous attributes of spring. Spring was the season that differentiated the most strongly from the others (i.e. summer and autumn), according to both community structure and interactions. In all five pans, spring was characterised by positive keystones. Positive synchronous associations can reflect mutualistic and facilitative interactions, but also parasitism, predation or similar niche preference. However, the direction of species correlations is not always obvious, for example, similar niche preference can manifest both as positive synchronous correlation due to coexistence or as negative due to competitive exclusion. Similarly, predation can display as positive synchronous associations when a prey attracts its predators to a habitat patch or as negative (both synchronous and time-shifted) when predators eliminate their preys (Barberán et al. 2011, Faust et al. 2018). Various positive keystone eOTUs abundant in spring were assigned to predatory flagellates such as Colpodellida (Mylnikov 2009) or intracellular parasitic taxa such as Cryptomycotina (Letcher et al. 2017) and Perkinsozoa (Mangot et al. 2011). These, together with the significant effect of *Daphnia magna* abundance on microeukaryotic community structure, suggest an important role of top-down controls in spring. Meanwhile, the spring-abundant positive actinobacterial keystones belonged to lineages (i.e. Luna1-A and acIV-C) that are characterised by very small cell sizes (<0.1 µm^3^) (Duda et al. 2012) and have been previously suggested to be grazing resistant (Tarao et al. 2009, Eckert et al. 2013), which might explain their coexistence with predatory eukaryotic taxa. Overall, the seasonal dynamics of actinobacterial keystones were similar to the seasonal dynamics described for this phylum in various other limnic systems (Eiler et al. 2012, Mikhailov et al. 2022).

Except for the eOTUs of Sós-ér, spring was also characterised by low species turnover, which could be the result of relative stability provided by deeper waters. The spring samples had a high abundance of a single *Choricystis* eOTU, a widely distributed freshwater picoeukaryotic green algal genus (Kulakova et al. 2020). These results agree with previous studies of soda pans of the region that showed that picoeukaryotic green algae are the most abundant members of the phytoplankton and have a specific seasonal trend with the highest abundances in winter-spring and the lowest in summer (Somogyi et al. 2011, 2016, 2022, Felföldi 2020, Szabó et al. 2020). The dominance of positive keystone taxa and the transitivity of positive correlations combined with low turnover of both eOTUs and bOTUs in the turbid pans suggests that spring was a period when community assembly was primarily ruled by trophic interactions between primary producer eukaryotic picoalgae (e.g. *Choricystis*), their parasites (e.g. Cryptomycotina), heterotrophic bacteria (e.g. Luna1 lineages, Burkholderiaceae, Balneolaceae) consuming algal exudates and debris, and flagellates (e.g. Colpodellida) predating on bacteria.

### The impact of local stressors

After the relatively stable and synchronous period in spring, the variation between microbial communities increased even for the turbid pans. This was driven by the higher and more variable concentrations of dissolved substances resulting from the lower water levels, which agrees with previous studies showing that environmental fluctuations induced by shrinking ecosystem size modulate community assembly processes and reduce stability (Bier et al. 2022). In the case of Kelemen-szék and Zab-szék, the decreasing water levels resulted in various desiccation events followed by refillments. Drying-rewetting cycles exert severe stress on microorganisms due to drastic changes in salt and nutrient content (Székely and Langenheder 2017, Schimel 2018, Truchy et al. 2020). While desiccation is common in soda pans of this region, not every pan dries out every year and it is not always the same pans that dry out (Boros et al. 2020, Szabó et al. 2020), making desiccation not a part of the regular seasonality, but rather a local stressor.

### Comparison of microeukaryotic and bacterial trends and stress response

Intense environmental fluctuation combined with increased growth rates due to summer warming and decreasing habitat size (i.e. shrinking water levels) were expected to stimulate microbial turnover (Vass et al. 2021, Bier et al. 2022). Accordingly, the turnover of microeukaryotic communities in the turbid pans increased relatively uniformly. Microeukaryotic turnover also increased substantially as a consequence of each drying-rewetting cycle, suggesting limited resilience of the microeukaryotic communities to such stressors. Simultaneously, the contribution of non-core eOTUs to the microeukaryotic communities of the drying pans was much higher than to those of the non-drying turbid pans and their relative abundance increased, particularly following drought events, supporting our hypothesis about non-core OTUs becoming more abundant in response to local stressors. A potential explanation for this phenomenon is that, despite the extensive soda pan-adapted microeukaryotic core community, drastic stress events like desiccation disrupt the species-sorting processes from the core community due to dispersal limitation or a lack of internal drought-resistant seed banks.

Meanwhile, the turnover of the bacterial communities of the turbid pans remained relatively similar through the study irrespective of desiccation, implying that bacteria were more resistant to the desiccation stress and the overall more extreme conditions of summer-autumn than microeukaryotes. Bacterial keystones of this period also suggest special adaptations to extreme conditions. For example, it has been shown for close relatives of the summer-autumn abundant Nitriliruptoraceae keystones that they can scavenge organic nitrogen even from strong nitrile bonds (Sorokin et al. 2009), allowing them versatility to overcome nitrogen limitation. The high number of negative bacterial keystones in summer-autumn also suggests that bacterial groups with different optima were dynamically outcompeting each other under the quickly changing conditions. The contribution of core4 bOTUs was also very high at every sampling time and site, suggesting that the core bacterial community of turbid pans not only contributed to the adaptation to seasonal changes, but was also highly resistant to extreme conditions. As it has been shown that drying-rewetting cycles have strong filtering effects on bacterial communities and dispersal is required for full recovery (Fazi et al. 2008, 2013, Székely and Langenheder 2017), the similar contribution of core4 bOTUs to the drying and non-drying turbid pans, as well as the uninterrupted high contribution of core4 bOTUs irrespective of drought events, suggests no dispersal limitation for bacteria between the studied pans. Recently, it has been suggested that waterbirds play an important role in dispersing both prokaryotes and microeukaryotes between soda pans (i.e. endozoochory) (Szabó et al. 2022), which implies that dispersal intensity would vary with bird visitation frequency. Although our study covered periods with different bird abundances (Boros et al. 2023), the absence of indications of bacterial dispersal limitation suggests that the bacterial core microbiome is highly dispersed between the pans by non-endozoochory dispersal such as wind or precipitation (Langenheder and Székely 2011).

The overall higher contribution of core bOTUs to keystone taxa than that of core eOTUs indicates more synchronised community trends for bacteria than for microeukaryotes. In general, microeukaryotic communities were more sensitive to the local stressors, probably both due to less physiological resistance and dispersal limitation. All in all, this supports our hypothesis regarding the lesser impact of local stress events on bacterial compared with microeukaryotic communities. Our results are also in agreement with studies suggesting that dispersal limitation and stochasticity are more important in shaping microeukaryotic communities, while selection processes are more prominent in bacterial community assembly and with studies that identified increased dispersal limitation during dry periods for microeukaryotic communities (Beisner et al. 2006, Wang et al. 2015, Logares et al. 2018, Chen et al. 2019, Mikhailov et al. 2022).

### Effect of seasonal trends and local stressors on microbial interactions

The networks generated in this study allowed for joint analyses of the interactions of microeukaryotes and bacteria. All networks had more positive correlations than negative, suggesting predominance of positive interactions in the communities. Previous studies demonstrated that positive associations are more common in ecosystems characterised by high abiotic stress due to a higher number of mutualistic interactions permitting species to exist in harsher environments than would otherwise be possible (Travis et al. 2005, Hernandez et al. 2021). However, the dependence on mutualism makes such communities sensitive to perturbations and reduces network stability, particularly in the case of low modularity networks (Hernandez et al. 2021). Apart from the dominance of positive correlations, there were clear differences in network topology and properties between the brown Sós-ér, the two drying (Kelemen-szék and Zab-szék) and the two non-drying turbid pans (Böddi-szék and Pan no.60). The networks of the latter pans reflected the most consistent and stable seasonal succession processes, with distinct hubs corresponding to different seasons (i.e. spring and summer-autumn) and a high number of mainly negative global (i.e. SSCC) time-shifted associations. For the drying pans, seasonal network modularity was less clear and dominance of synchronous local (i.e. LS) associations was the most characteristic, indicating lesser importance of community dynamics overarching the entire study period. Meanwhile, the networks of Sós-ér had three hubs, with one corresponding to the late summer period characterised by *Nodularia* bloom. Overall, the interaction networks of the soda pans reflected low community stability in these high stress habitats that was further exacerbated by local stress events, such as drying-rewetting cycles or cyanobacterial blooms.

## Conclusions

By integrating network analyses, assessment of keystone taxa and the consideration of microeukaryotic and bacterial communities separately, we gained novel and comprehensive insights into the unique seasonal dynamics of shallow soda lakes. Our findings revealed that despite significant environmental changes and subsequent community shifts, the studied soda pans were primarily inhabited by a common core microbiome and share certain characteristics of their seasonal trends. However, the extent of the shared microbiome was curtailed among pans of different habitat subtype (i.e. brown or turbid pan) and local stress events like desiccation and refillment, modified common seasonal patterns. In general, stable environmental conditions during spring fostered stable microbial communities governed by trophic interactions. Conversely, the dynamic changes of summer and early autumn, coupled with local stress events, exerted strong selective pressure that instigated varied response mechanisms in microeukaryotic and bacterial communities. Species recruitment from the core community played a key role in the adaptation to seasonal changes for both microeukaryotes and bacteria, while for microeukaryotes response to stress events involved a large part of the non-core communities, suggesting the influence of dispersal limitation in their recovery. By contrast, bacterial communities were, to a great extent, resistant to stressors and adapted to extreme conditions through species sorting from the core community and competitive exclusion. To the best of our knowledge, this provides the first evidence in extreme aquatic habitats in support of the hypothesis that microeukaryotic communities exhibit higher sensitivity to stress events compared with bacteria. Furthermore, our study underscores the importance of separately considering microeukaryotic and bacterial communities when assessing the impacts of local stressors. This is particularly relevant as events like desiccation and refillment become more frequent in shallow aquatic ecosystems due to climate change.

## Supplementary Material

fiad095_Supplemental_Files

## Data Availability

Raw sequence reads can be accessed at the NCBI SRA through BioProject ID PRJNA272672 and Biosample IDs SAMN32532920-SAMN32532982.
