# Supplementary material for: Contrasting response of microeukaryotic and bacterial communities to the interplay of seasonality and local stressors in shallow soda lakes
Source: FEMS Microbiol Ecol. 2023 Aug 16;99(9):fiad095. doi: 10.1093/femsec/fiad095 (PMC10449373; doi:10.1093/femsec/fiad095)
Supplement: fiad095_Supplemental_Files [file fiad095_supplemental_files.zip › Supp_data _ZMarton_minor_revision.docx]

**Supplementary material**

**Contrasting Response of Microeukaryotic and Bacterial Communities to the Interplay of Seasonality and Local Stressors in Shallow Soda Lakes**

Zsuzsanna Márton ^1,2,3^, Bianka Csitári^3,6,8^, Tamás Felföldi^1,4^, Ferenc Jordán^7^, András Hidas^1,3^, Attila Szabó^1,5#^ & Anna J. Székely^5,8#^

^1^Institute of Aquatic Ecology, Centre for Ecological Research, Budapest, Hungary

^2^National Multidisciplinary Laboratory for Climate Change, Centre for Ecological Research, Budapest, Hungary

^3^Doctoral School of Environmental Sciences, ELTE Eötvös Loránd University, Budapest, Hungary

^4^ELTE Eötvös Loránd University, Budapest, Hungary

^5^Swedish University of Agricultural Sciences, Uppsala, Sweden

^6^Karolinska Institutet, Stockholm, Sweden

^7^Department of Chemistry, Life Sciences and Environmental Sustainability, University of Parma, Parma, Italy

^8^Uppsala University, Uppsala, Sweden

^#^These authors contributed equally to this work

## Corresponding author

Anna J. Székely

Swedish University of Agricultural Sciences, Dep. of Aquatic Sciences and Assessment, Box 7050, 75007 Uppsala, Sweden. E-mail address: [anna.szekely@slu.se](mailto:anna.szekely@slu.se)

This file includes the following text, tables and figures:

Text S1-S3

Table S2-S4

Figure S1-S2

**Text S1** 18S and 16S rRNA gene amplicon sequencing

Eukaryotic primers 574*F (CGGTAAYTCCAGCTCYAV) and 1132R (CCGTCAATTHCTTYAART)^1^ and prokaryotic primers 341F (CCTACGGGNGGCWGCAG)^2^, 805NR (GACTACHVGGGTATCTAATCC)^3^ were used for the polymerase chain reactions. To decrease the stochastic effect of the reaction, all PCR amplification was performed in duplicates in 20 µL, which contained 4 µL of 5x Q5 reaction buffer, 2 µL of dNTP (2 mM), 0.2 µL of Q5 High Fidelity DNA polymerase (2 U/µL) (New England Biolabs), 0.5 µL of each primer (10 µM), 11.8 µL of nuclease free water and 1 µL of template DNA. The following thermal cycle conditions were used for 18S rRNA gene amplification: initial denaturation at 98 °C for 1 min 10 sec, followed by 20 cycles (annealing at 51 °C for 30 sec, extension at 72 °C for 30 sec) and a final elongation step at 72 °C for 2 min. The following thermal cycle conditions were used for 16S rRNA gene amplification: initial denaturation at 98 °C for 40 sec, followed by 20 cycles (annealing at 48 °C for 30 sec, extension at 72 °C for 30 sec) and a final elongation step at 72 °C for 2 min. Amplicons were pooled before purification with magnetic beads (Agencourt AMPure XP PCR Purification, 2013).  To prepare libraries for Illumina sequencing, primers were prolonged by Illumina handles and index primers. The second PCR reaction contained 4 µL of 5x Q5 reaction buffer, 2 µL of dNTP (2 mM), 0.2 µL of Q5 High Fidelity DNA polymerase, 1 µL of each index primer (5 µM), 9.8 µL of nuclease free water and 1 µL of template from the first PCR. The following thermal cycle was used for both eukaryote and prokaryote specific reaction: initial denaturation 98 °C for 40 sec, followed by 15 cycles of denaturation 98 °C for 10 sec, annealing at 66 °C for 30 sec, extension at 72 °C for 30 sec/kb and the final extension at 72°C for 2 min. Amplicons were purified again with magnetic beads (Agencourt AMPure) as described previously. Quantification of the libraries were carried out using a PicoGreen assay (Quant-iT PicoGreen dsDNA Assay Kit, Invitrogen). Sequencing was performed at the SciLifeLab (Uppsala, Sweden) on an Illumina MiSeq platform (Illumina Inc, San Diego, CA, USA).

**Text S2 Bioinformatic analysis of the sequencing data**

Bioinformatic analysis of the sequence reads were carried out with mothur v1.41.1^4^ using the MiSeq SOP (http://www.mothur.org/wiki/MiSeq_SOP downloaded at 9th July 2018). The deltaq parameter of the ’make.contigs’ command was adjusted to 10 for additional quality filtering to eliminate sequencing errors. Primers were removed from the start and the end of the sequences and singletons were also removed from the dataset according to ^5^. For the alignment of sequence reads the ARB-SILVA SSU Ref NR 132 reference database^6^ was used. Denoising was performed using mothur’s pre.cluster command using the default algorithm (Huse et al., 2010) and applying the suggested 4 bp difference cutoff. Chimeras were identified and removed using the mothur implemented version of VSEARCH. Operational taxonomic units (OTUs) were assigned at 99% similarity threshold levels with the OptiClust algorithm^7^. Taxonomic assignment of the 18S rRNA gene OTUs was carried out using the PR^2^ v4.10 reference database^8^ with a minimum bootstrap confidence score of 80 and applying 1000 iterations. For the 16S rRNA gene amplicon set the TaxAss software^9^ was used for taxonomic classification applying default parameters and using the FreshTrain (2018 April 30 release) and ARB-SILVA SSU Ref NR 132 databases as reference. Bacterial OTUs assigned to non-primer specific taxonomic groups (e.g. Archaea, chloroplasts, mitochondria, unknown) and microeukaryotic OTUs assigned to taxa Metazoa, Streptophyta, Basidiomycota and Ascomycota (due to the prefiltration through a 40 µm pore sized mesh of the water samples) were removed from the dataset. The 7th sampling time of Pan no. 60 was discarded from the 18S rRNA gene amplicon dataset due to low number of high-quality sequences. For statistical analyses, reads were subsampled to the read number of the sample having the lowest sequence count (62 samples in the 18S rRNA amplicon set, n = 2407 and 63 samples in the 16S rRNA amplicon set, n = 3188).

**Text S3 Measured environmental parameters**

Water temperature increased from 13.7 °C to 27.8 °C during spring (mean: 20.0 °C) and decreased from 24.8 °C to 7.1 °C during autumn (mean: 16.3 °C), while in summer varied between 19.6 and 30.9 °C (mean: 25.2 °C). Salinity values varied between the subsaline (min. 0.9 g/L) and mesosaline (max. 27.8 g/L) categories with the majority of samples (54 out of 63) being hyposaline (3-20 g/L)^10^. The values of DOC, TN and TP varied between 10 and 3341 mg/L, 1.5 and 25.7 mg/L and 0.5 and 25 mg/L, respectively. The pH value remained alkaline throughout the study period in all pans with an average of 9.5 pH and varying from 8.5 to 10.0. The pans were aerobic at each sampling time (O_2_ saturation >79%) and often over-saturated (O_2_ saturation >100%). Chlorophyll a concentration ranged between 1.8 and 696.7 µg/L, with an average of 204.1 µg/L with both the lowest and highest chlorophyll a values measured in Pan no. 60 (Figure S1).


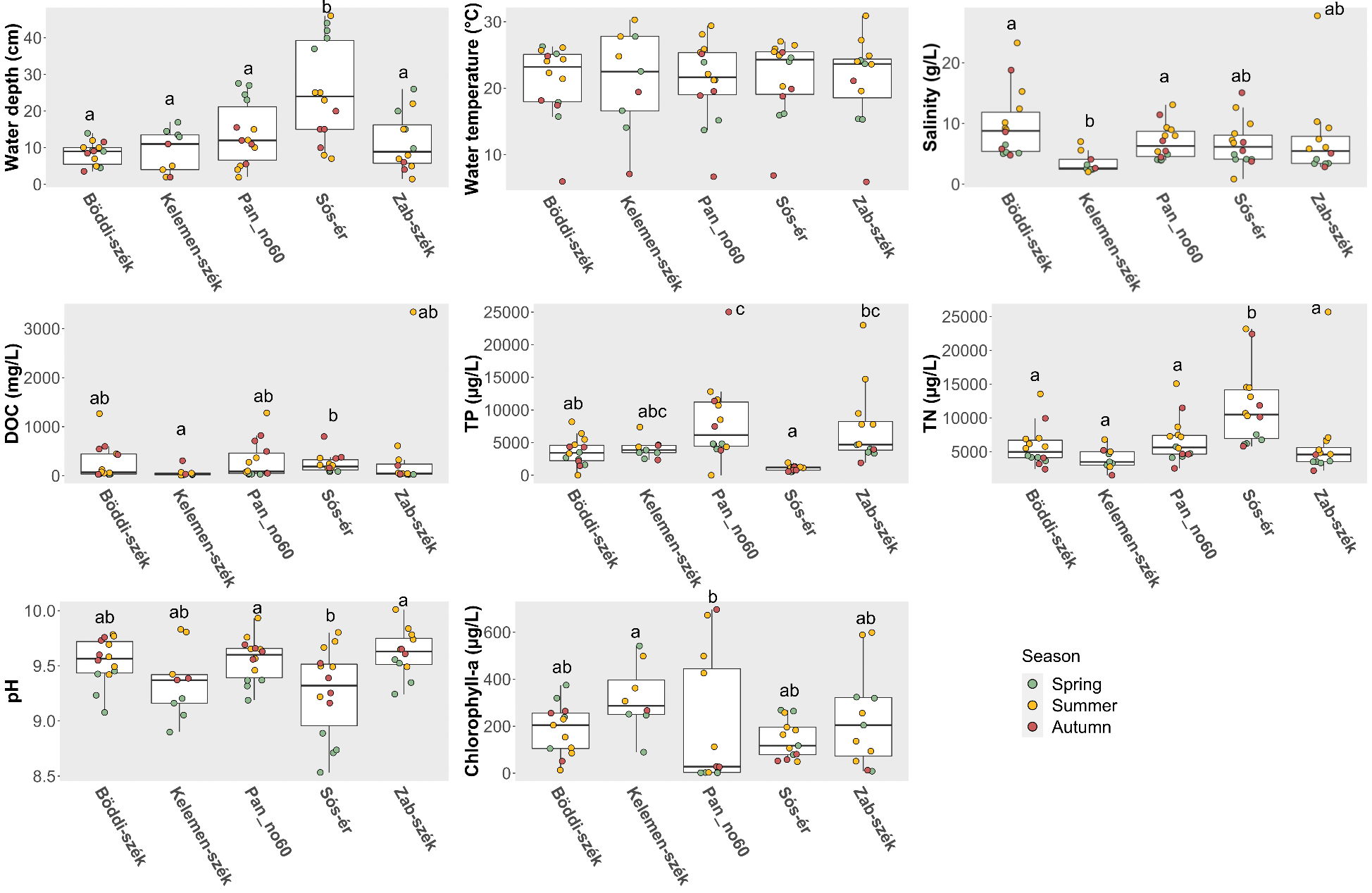


**Figure S1** Environmental parameters of the soda pans. Different letters within soda pans indicate statistically significant differences at a significant level of p < 0.05.


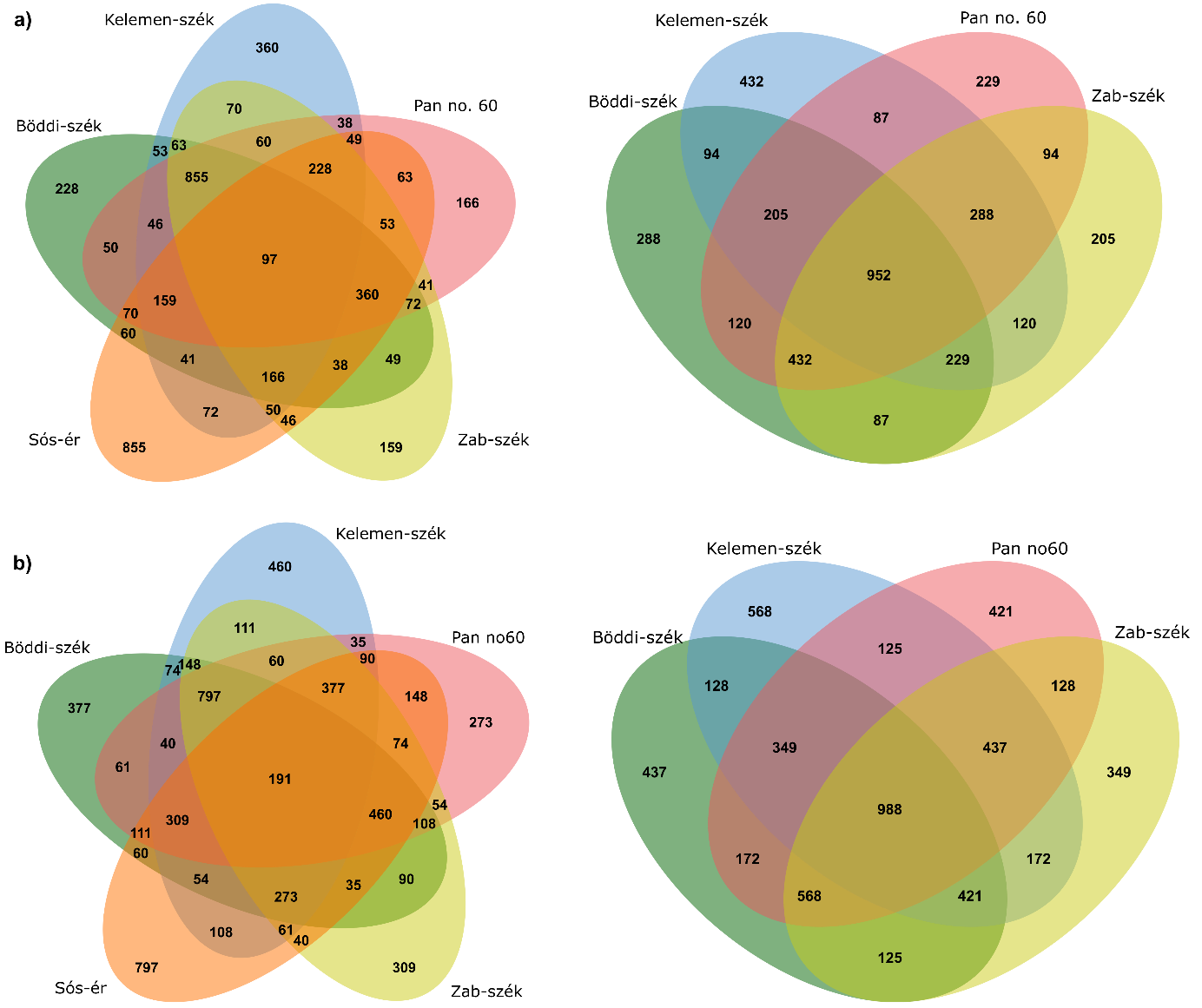


**Figure S2** Venn diagrams showing the numbers of shared and unique OTUs between all the five pans and the four turbid type of soda pans: a) Microeukaryotic OTUs, b) Bacterial OTUs (Venn diagrams were generated using jvenn^11^, to visualize the core microbial community of the pans)


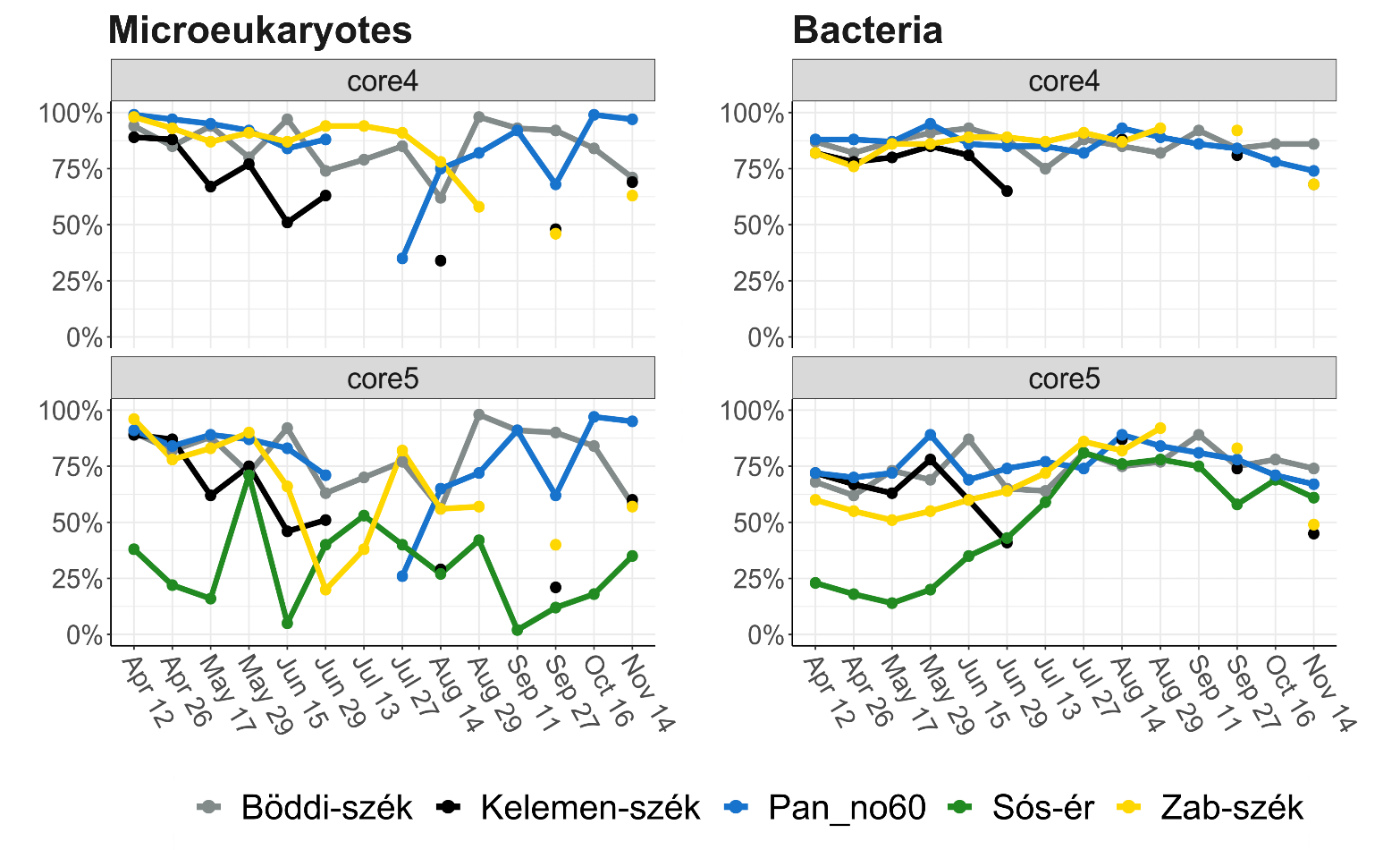


**Figure S3** Relative abundance of the core4 and core5 OTUs in the studied pans during the study period

**Table S2** Abbreviated names, mean, minimum and maximum relative abundance of the microeukaryotic genera and bacterial clades. Taxa that are contain OTUs of the core4 or core5 community are also indicated.

**Microeukaryotes**

| **Abbreviation** | **Genus/clade** | **Core** | **Böddi-szék** | | **Pan no. 60** | | **Kelemen-szék** | | **Zab-szék** | | **Sós-ér** | | |
| --- | --- | --- | --- | --- | --- | --- | --- | --- | --- | --- | --- | --- | --- |
|  |  |  | Mean | Min-Max | Mean | Min-Max | Mean | Min-Max | Mean | Min-Max | | Mean | Min-Max |
| Ad | *Andalucia* | 4, 5 |  |  | 0.012 | 0-0.158 |  |  |  |  | |  |  |
| An | *Anomoeoneis* | 4, 5 |  |  |  |  | 0.011 | 0-0.090 | 0.011 | 0-0.099 | | 0.021 | 0-0.164 |
| Ca | Chlamydomonadales_X_unclassified | 4, 5 |  |  |  |  |  |  | 0.021 | 0-0.241 | |  |  |
| Cd | Chrysophyceae_Clade-D_X | 4, 5 |  |  |  |  |  |  |  |  | | 0.011 | 0-0.128 |
| Ce | Cercozoa_unclassified | 4, 5 | 0.005 | 0-0.056 |  |  |  |  | 0.025 | 0-0.134 | |  |  |
| Cf | Chrysophyceae_Clade-F_X | 4, 5 |  |  |  |  |  |  |  |  | | 0.013 | 0-0.126 |
| Ch | *Choricystis* | 4, 5 | 0.211 | 0.002-0.575 | 0.115 | 0.003-0.624 | 0.363 | 0-0.825 | 0.304 | 0.007-0.864 | | 0.051 | 0-0.225 |
| Cl | *Chloroparva* | 4, 5 | 0.041 | 0.005-0.195 | 0.192 | 0.018-0.472 |  |  | 0.024 | 0.004-0.101 | | 0.013 | 0-0.097 |
| Cr | Chlorophyta_unclassified | 4, 5 | 0.013 | 0-0.137 |  |  |  |  | 0.011 | 0-0.062 | | 0.026 | 0-0.231 |
| Ct | Chytridiomycetes_unclassified | 4 |  |  |  |  |  |  | 0.007 | 0-0.093 | |  |  |
| Cu | Chlorellales_X_unclassified | 4, 5 | 0.091 | 0.003-0.307 | 0.163 | 0.009-0.419 | 0.035 | 0-0.296 | 0.126 | 0-0.651 | | 0.138 | 0-0.538 |
| Cy | Chrysophyceae_X_unclassified | 4, 5 |  |  |  |  |  |  | 0.034 | 0-0.230 | |  |  |
| Di | *Diacronema* | 4, 5 |  |  |  |  |  |  | 0.008 | 0-0.099 | | 0.033 | 0-0.231 |
| Fu | Fungi_unclassified | 4, 5 | 0.005 | 0-0.051 | 0.013 | 0-0.141 |  |  |  |  | | 0.009 | 0-0.098 |
| Ha | *Halocafeteria* | - |  |  |  |  |  |  | 0.011 | 0-0.059 | |  |  |
| Hl | *Halteria* | - |  |  |  |  | 0.069 | 0-0.592 |  |  | | 0.007 | 0-0.051 |
| Hn | *Hanusia* | - |  |  |  |  |  |  |  |  | | 0.009 | 0-0.067 |
| Hp | *Hoplorhynchus* | 4 |  |  |  |  |  |  |  |  | | 0.007 | 0-0.094 |
| Ht | Halteriidae_X | 4, 5 |  |  |  |  |  |  |  |  | | 0.021 | 0-0.167 |
| Ko | *Komma* | - |  |  |  |  |  |  |  |  | | 0.051 | 0-0.537 |
| Ma | *Marvania* | 4, 5 | 0.012 | 0-0.073 |  |  | 0.048 | 0-0.375 |  |  | |  |  |
| Na | *Nannochloris* | 4, 5 | 0.046 | 0-0.439 | 0.019 | 0-0.107 |  |  |  |  | |  |  |
| Ni | *Nitzschia* | 4, 5 | 0.039 | 0-0.300 |  |  | 0.051 | 0-0.292 | 0.031 | 0-0.275 | |  |  |
| Nn | *Nannochloropsis* | 4, 5 |  |  | 0.067 | 0-0.626 |  |  |  |  | | 0.005 | 0-0.053 |

**Microeukaryotes (cont.)**

| **Abbreviation** | **Genus/clade** | **Core** | **Böddi-szék** | | **Pan no. 60** | | **Kelemen-szék** | | **Zab-szék** | | **Sós-ér** | | |
| --- | --- | --- | --- | --- | --- | --- | --- | --- | --- | --- | --- | --- | --- |
|  |  |  | Mean | Min-Max | Mean | Min-Max | Mean | Min-Max | Mean | Min-Max | | Mean | Min-Max |
| No | Novel-clade-2_X | 4, 5 |  |  |  |  |  |  | 0.032 | 0-0.325 | |  |  |
| Nv | Novel-Gran-6_X | 4, 5 |  |  | 0.011 | 0-0.103 |  |  |  |  | |  |  |
| Oc | Ochrophyta_unclassified | 4, 5 |  |  | 0.005 | 0-0.064 |  |  |  |  | | 0.029 | 0-0.067 |
| Op | Opisthokonta_unclassified | 4, 5 |  |  | 0.019 | 0-0.241 |  |  |  |  | | 0.017 | 0-0.227 |
| Pa | *Paraphysomonas* | 4, 5 |  |  | 0.009 | 0-0.122 |  |  |  |  | | 0.037 | 0-0.268 |
| Pe | Perkinsida_XXX | - |  |  | 0.034 | 0-0.417 |  |  |  |  | |  |  |
| Pi | *Pirsonia*_unclassified | - | 0.006 | 0-0.085 | 0.008 | 0-0.101 | 0.014 | 0-0.129 |  |  | |  |  |
| Pl | Platyophryida_unclassified | - |  |  |  |  |  |  | 0.005 | 0-0.061 | |  |  |
| Pn | *Pirsonia* | 4, |  |  |  |  |  |  |  |  | | 0.019 | 0-0.259 |
| Pr | *Prorocentrum* | - |  |  |  |  | 0.032 | 0-0.167 |  |  | | 0.011 | 0-0.094 |
| Ps | Pseudodendromonadales_XX | 4, 5 |  |  |  |  |  |  |  |  | | 0.006 | 0-0.69 |
| Py | *Pythium* | - |  |  |  |  |  |  |  |  | | 0.012 | 0-0.103 |
| Ra | Raphid-pennate_unclassified | 4, 5 |  |  |  |  |  |  | 0.005 | 0-0.059 | |  |  |
| Se | Sessilida_unclassified | 4, 5 |  |  |  |  | 0.006 | 0-0.052 |  |  | |  |  |
| So | Sordariomycetes_unclassified | 4, 5 |  |  |  |  |  |  |  |  | | 0.049 | 0-0.557 |
| Sp | *Spumella* | 4, 5 | 0.037 | 0-0.408 | 0.063 | 0-0.793 |  |  | 0.013 | 0-0.153 | |  |  |
| St | Stramenopiles_unclassified | 4, 5 |  |  | 0.007 | 0-0.097 |  |  | 0.026 | 0-0.306 | |  |  |
| Su | *Surirella* | 4, 5 | 0.005 | 0-0.057 |  |  |  |  |  |  | |  |  |
| Te | *Tetracystis* | - |  |  |  |  |  |  |  |  | | 0.007 | 0-0.091 |
| Tr | *Tremula* | 4, 5 |  |  | 0.005 | 0-0.074 |  |  |  |  | |  |  |
| Vo | *Vorticella* | 4, 5 |  |  |  |  |  |  |  |  | | 0.008 | 0-0.116 |
| Wi | *Wislouchiella* | 4 |  |  |  |  | 0.027 | 0-0.203 |  |  | |  |  |

**Bacteria**

| **Abbreviation** | **Genus/clade** | **Core** | **Böddi-szék** | | **Pan no. 60** | | **Kelemen-szék** | | **Zab-szék** | | **Sós-ér** | | |
| --- | --- | --- | --- | --- | --- | --- | --- | --- | --- | --- | --- | --- | --- |
|  |  |  | Mean | Min-Max | Mean | Min-Max | Mean | Min-Max | Mean | Min-Max | | Mean | Min-Max |
| Ab | Absconditabacteriales_SR1 | 4, 5 |  |  |  |  |  |  |  |  | |  |  |
| Ai | Acidithiobacillaceae | 4 |  |  |  |  |  |  | 0.007 | 0-0.089 | | 0.007 | 0-0.101 |
| ac | acIII-A | 4, 5 | 0.026 | 0.032-0.059 | 0.034 | 0.017-0.095 | 0.064 | 0.018-0.232 | 0.043 | 0.013-0.157 | | 0.025 | 0.002-0.086 |
| aV | acIV-C | 4, 5 | 0.011 | 0-0.054 |  |  |  |  |  |  | |  |  |
| Ac | Actinobacteria | 4, 5 |  |  |  |  |  |  |  |  | | 0.013 | 0-0.056 |
| Al | *Algoriphagus* | 4, 5 |  |  |  |  |  |  |  |  | | 0.012 | 0-0.061 |
| ba | bacII-A | 4 |  |  |  |  |  |  |  |  | | 0.037 | 0-0.216 |
| bc | bacV |  |  |  |  |  |  |  |  |  | | 0.015 | 0-0.064 |
| Ba | Balneolaceae | 4, 5 | 0.016 | 0.002-0.068 | 0.008 | 0-0.059 |  |  |  |  | |  |  |
| Bu | Burkholderiaceae | 4, 5 |  |  |  |  | 0.026 | 0-0.143 |  |  | | 0.042 | 0-0.165 |
| Cc | Candidatus_Campbellbacteria | 4, 5 |  |  |  |  | 0.008 | 0-0.051 |  |  | |  |  |
| Ce | *Cecembia* | 4, 5 | 0.005 | 0-0.066 |  |  |  |  |  |  | |  |  |
| Cr | Cryomorphaceae | 4, 5 | 0.009 | 0-0.071 |  |  |  |  |  |  | |  |  |
| Cy | *Cyanobium*_PCC-6307 | 4, 5 |  |  | 0.119 | 0.002-0.281 |  |  | 0.021 | 0-0.102 | | 0.008 | 0-0.067 |
| Cl | Cyclobacteriaceae | 4, 5 | 0.005 | 0-0.066 |  |  |  |  |  |  | | 0.011 | 0-0.058 |
| Er | Erysipelotrichaceae_UCG-004 | 4, 5 |  |  |  |  |  |  |  |  | | 0.093 | 0-0.318 |
| Fl | Flavobacteriaceae | 4, 5 |  |  |  |  |  |  |  |  | | 0.042 | 0-0.328 |
| Ge | Gemmatimonadetes | 4, 5 |  |  | 0.038 | 0-0.224 |  |  |  |  | |  |  |
| Hy | *Hydrogenophaga* | 4, 5 |  |  |  |  |  |  |  |  | | 0.027 | 0-0.093 |
| Iu | *Ilumatobacter* | 4, 5 |  |  | 0.025 | 0.001-0.087 |  |  |  |  | |  |  |
| Il | Ilumatobacteraceae | 4, 5 | 0.036 | 0.011-0.086 | 0.021 | 0.002-0.054 | 0.029 | 0.006-0.106 | 0.034 | 0-0.168 | |  |  |
| Iz | Izimaplasmatales | 4, 5 | 0.011 | 0.001-0.052 |  |  |  |  |  |  | |  |  |
| Ki | Kiritimatiellaeota | 4, 5 |  |  | 0.053 | 0-0.224 |  |  |  |  | | 0.006 | 0-0.067 |
| Lu | Luna1-A | 4, 5 | 0.059 | 0.009-0.195 | 0.014 | 0-0.059 | 0.075 | 0.017-0.186 |  |  | | 0.071 | 0-0.319 |
| Mt | Methylophilaceae | 4, 5 | 0.024 | 0.007-0.063 |  |  |  |  |  |  | | 0.035 | 0-0.067 |

**Bacteria**

| **Abbreviation** | **Genus/clade** | **Core** | **Böddi-szék** | | **Pan no. 60** | | **Kelemen-szék** | | **Zab-szék** | | **Sós-ér** | | |
| --- | --- | --- | --- | --- | --- | --- | --- | --- | --- | --- | --- | --- | --- |
|  |  |  | Mean | Min-Max | Mean | Min-Max | Mean | Min-Max | Mean | Min-Max | | Mean | Min-Max |
| Me | *Methylotenera* | 4, 5 |  |  |  |  | 0.024 | 0.005-0.067 |  |  | |  |  |
| Mi | Microscillaceae | 4, 5 |  |  |  |  | 0.012 | 0-0.061 |  |  | |  |  |
| Ni | Nitriliruptoraceae | 4, 5 | 0.092 | 0.032-0.201 | 0.085 | 0.020-0.292 | 0.019 | 0.002-0.063 | 0.083 | 0.018-0.324 | | 0.055 | 0-0.295 |
| No | *Nodularia*_PCC-9350 | 4, 5 |  |  |  |  |  |  |  |  | | 0.058 | 0-0.404 |
| Ox | Oxyphotobacteria | 4, 5 | 0.013 | 0-0.079 |  |  | 0.035 | 0-0.199 | 0.017 | 0-0.051 | |  |  |
| Pa | Parcubacteria | 4, 5 |  |  |  |  |  |  | 0.037 | 0-0.134 | | 0.008 | 0-0.114 |
| Pi | Phycisphaeraceae | 4 |  |  | 0.024 | 0-0.130 | 0.014 | 0-0.115 |  |  | | 0.006 | 0-0.066 |
| Pl | *Planktosalinus* | 4, 5 | 0.044 | 0-0.152 | 0.019 | 0-0.141 |  |  | 0.018 | 0-0.056 | | 0.004 | 0-0.057 |
| Rh | *Rhodobaca* | 4, 5 | 0.024 | 0-0.082 |  |  |  |  | 0.016 | 0-0.058 | |  |  |
| Ru | *Ruminiclostridium*_1 | 4 | 0.011 | 0-0.088 |  |  |  |  | 0.013 | 0-0.069 | |  |  |
| Sa | Saprospiraceae | 4, 5 | 0.014 | 0-0.064 | 0.023 | 0-0.113 | 0.008 | 0-0.073 |  |  | | 0.005 | 0-0.052 |
| Sp | Sphingobacteriales | 4, 5 |  |  | 0.006 | 0-0.063 | 0.009 | 0-0.052 |  |  | | 0.012 | 0-0.085 |
| Sp | Sporichthyaceae | 4 | 0.019 | 0.003-0.053 |  |  |  |  | 0.028 | 0.006-0.052 | |  |  |
| Sy | *Synechococcus*_MBIC10613 | 4, 5 | 0.036 | 0-0.219 | 0.028 | 0.001-0.132 |  |  |  |  | |  |  |

**Table S3** Mantel test results between the microeukaryotic and bacterial communities, and environmental variables and zooplankton species based on Spearman’s rank correlation (rho = 1 “strong positive correlation”, rho = -1 “strong negative correlation”) (bold: significant)

| **Mantel test between** | | **Spearman’s correlation coefficient (rho)** | **Significance of the test (p)** |
| --- | --- | --- | --- |
| **Microeukaryotes** | Water depth | -0.004 | 0.470 |
|  | Water temperature | -0.022 | 0.617 |
|  | **Salinity** | **0.190** | **0.005** |
|  | **DOC** | **0.313** | **0.001** |
|  | **TP** | **0.248** | **0.001** |
|  | TN | 0.188 | 0.007 |
|  | pH | 0.066 | 0.138 |
|  | Chlorophyll-a | 0.051 | 0.179 |
|  | *Daphnia magna* | -0.090 | 0.864 |
|  | *Daphnia atkinsoni* | -0.057 | 0.725 |
|  | *Moina brachiata* | 0.003 | 0.446 |
|  | *Arctodiaptomus spinosus* | 0.021 | 0.331 |
|  | *Arctodiaptomus bacilifer* | -0.108 | 0.910 |
|  | *Megacyclops viridis* | -0.112 | 0.895 |
|  | Copepoda | -0.019 | 0.571 |
|  | Cladocera | 0.007 | 0.451 |
| **Bacteria** | Water depth | 0.101 | 0.068 |
|  | **Water temperature** | **0.121** | **0.046** |
|  | Salinity | 0.079 | 0.101 |
|  | **DOC** | **0.135** | **0.045** |
|  | **TP** | **0.194** | **0.003** |
|  | TN | 0.176 | 0.012 |
|  | pH | 0.075 | 0.129 |
|  | Chlorophyll-a | 0.075 | 0.139 |
|  | *Daphnia magna* | -0.048 | 0.712 |
|  | *Daphnia atkinsoni* | 0.017 | 0.395 |
|  | *Moina brachiata* | -0.056 | 0.759 |
|  | ***Arctodiaptomus spinosus*** | **0.129** | **0.044** |
|  | *Arctodiaptomus bacilifer* | -0.123 | 0.943 |
|  | *Megacyclops viridis* | -0.064 | 0.743 |
|  | Copepoda | 0.102 | 0.084 |
|  | Cladocera | -0.050 | 0.734 |

**Table S4** Differences of planktonic microbial communities from the five soda pans comparing the three studied seasons. (PERMANOVA test results; number of * indicates the statistical significance with p. 0 ’***. 0.001 ’**’. 0.01 ’*’. 0.05 ’**^.^**’. 0.1 ’ ’. 1)

|  | **Spring/ Summer**  **(R^2^)** | **Spring/ Summer**  **(p)** | **Spring/ Autumn**  **(R^2^)** | **Spring/ Autumn**  **(p)** | **Summer/ Autumn**  **(R^2^)** | **Summer/ Autumn**  **(p)** |
| --- | --- | --- | --- | --- | --- | --- |
| **Micoreukaryotes** | 0.129 | 0.001*** | 0.185 | 0.001*** | 0.043 | 0.014* |
| **Bacteria** | 0.102 | 0.001*** | 0.124 | 0.001*** | 0.039 | 0.045* |

**Table S5** Impact of pan identity and seasonality on the structure of microeukaryotic and bacterial communities

(PERMANOVA test results; number of * indicates the statistical significance with p. 0 ’***. 0.001 ’**’. 0.01 ’*’. 0.05 ’**^.^**’. 0.1 ’ ’. 1)

|  |  |  | **Pan identity**  (R^2^) | **Pan identity**  (p) | **Seasonality**  (R^2^) | **Seasonality**  (p) | **Pan identity***  **Seasonality**  (R^2^) | **Pan identity***  **Seasonality**  (p) |
| --- | --- | --- | --- | --- | --- | --- | --- | --- |
| **All pans** | **Microeukaryotes** | All OTUs | 0.186 | **0.001***** | 0.144 | **0.001***** | 0.162 | **0.001***** |
|  |  | Core5 | 0.178 | **0.001***** | 0.151 | **0.001***** | 0.147 | **0.001***** |
|  |  | Non-core5 | 0.153 | **0.001***** | 0.069 | **0.001***** | 0.173 | **0.001***** |
|  | **Bacteria** | All OTUs | 0.255 | **0.001***** | 0.115 | **0.001***** | 0.163 | **0.001***** |
|  |  | Core5 | 0.188 | **0.001***** | 0.176 | **0.001***** | 0.127 | **0.007**** |
|  |  | Non-core5 | 0.151 | **0.001***** | 0.125 | **0.001***** | 0.148 | **0.001***** |
| **Turbid pans** | **Microeukaryotes** | All turbid pan OTUs | 0.144 | **0.001***** | 0.206 | **0.001***** | 0.132 | **0.006**** |
|  |  | Core4 | 0.145 | **0.001***** | 0.226 | **0.001***** | 0.124 | **0.015*** |
|  |  | Non-core4 | 0.129 | **0.001***** | 0.084 | **0.001***** | 0.164 | **0.001***** |
|  | **Bacteria** | All turbid pan OTUs | 0.177 | **0.001***** | 0.160 | **0.001***** | 0.137 | **0.003**** |
|  |  | Core4 | 0.177 | **0.001***** | 0.171 | **0.001***** | 0.129 | **0.012*** |
|  |  | Non-core4 | 0.167 | **0.001***** | 0.108 | **0.001***** | 0.159 | **0.001***** |

**Table S6** Impact of pan identity and seasonality on the Bray-Curtis dissimilarity index of microeukaryotic and bacterial communities (ANOVA test results; number of * indicates the statistical significance with p. 0 ’***. 0.001 ’**’. 0.01 ’*’. 0.05 ’**^.^**’. 0.1 ’ ’. 1)

| **Bray-Curtis dissimilarity** | **Pan identity**  (Df) | **Pan identity**  (SS) | **Pan identity**  (F) | **Pan identity**  (p) | **Seasonality**  (Df) | **Seasonality**  (SS) | **Seasonality**  (F) | **Seasonality**  (p) | **Pan identity***  **Seasonality**  (Df) | **Pan identity***  **Seasonality**  (SS) | **Pan identity***  **Seasonality**  (F) | **Pan identity***  **Seasonality**  (p) |
| --- | --- | --- | --- | --- | --- | --- | --- | --- | --- | --- | --- | --- |
| **Microeukaryotes** | 4 | 0.235 | 2.646 | **0.047*** | 2 | 0.603 | 13.538 | **> 0.001***** | 8 | 0.508 | 2.854 | **0.013*** |
| **Bacteria** | 4 | 0.156 | 2.734 | **0.041*** | 2 | 0.266 | 9.533 | **> 0.001***** | 8 | 0.249 | 2.239 | **0.043*** |

**Table S7** General network properties generated using the NetworkAnalyzer plugin of Cytoscape v3.8.2.

a) Synchronous b) Time-shifted. (* Number of edges = Number of negative correlations + Number of positive correlations)

| **a)** | **Pan** | **Number of nodes** | **Number of edges*** | **Average number of neighbours** | **Density** | **Number of negative correlations** | **Number of positive correlations** |
| --- | --- | --- | --- | --- | --- | --- | --- |
|  | **Böddi-szék** | 199 | 1417 | 14.24 | 0.07 | 506 | 911 |
|  | **Kelemen-szék** | 170 | 2672 | 31.44 | 0.19 | 1020 | 1652 |
|  | **Pan no. 60** | 176 | 848 | 9.88 | 0.06 | 304 | 544 |
|  | **Sós-ér** | 139 | 314 | 5.17 | 0.05 | 53 | 261 |
|  | **Zab-szék** | 212 | 1839 | 17.35 | 0.08 | 549 | 990 |
| **b)** |  |  |  |  |  |  |  |
|  | **Böddi-szék** | 202 | 2246 | 22.24 | 0.11 | 911 | 1335 |
|  | **Kelemen-szék** | 147 | 689 | 9.37 | 0.06 | 207 | 482 |
|  | **Pan no. 60** | 182 | 1304 | 14.55 | 0.08 | 508 | 796 |
|  | **Sós-ér** | 99 | 153 | 4.03 | 0.07 | 12 | 141 |
|  | **Zab-szék** | 156 | 535 | 7.08 | 0.05 | 85 | 450 |

**Reference of the supplementary Texts and Figure**

1. Hugerth, L. W. *et al.* Systematic design of 18S rRNA gene primers for determining eukaryotic diversity in microbial consortia. *PLoS One* **9**, (2014).
2. Herlemann, D. P. R. *et al.* Transitions in bacterial communities along the 2000 km salinity gradient of the Baltic Sea. *ISME Journal* **5**, 1571–1579 (2011).
3. Apprill, A., Mcnally, S., Parsons, R. & Weber, L. Minor revision to V4 region SSU rRNA 806R gene primer greatly increases detection of SAR11 bacterioplankton. *Aquatic Microbial Ecology* **75**, 129–137 (2015).
4. Schloss, P. D. *et al.* Introducing mothur: Open-source, platform-independent, community-supported software for describing and comparing microbial communities. *Appl Environ Microbiol* (2009) doi:10.1128/AEM.01541-09.
5. Kunin, V., Engelbrektson, A., Ochman, H. & Hugenholtz, P. Wrinkles in the rare biosphere: Pyrosequencing errors can lead to artificial inflation of diversity estimates. *Environ Microbiol* (2010) doi:10.1111/j.1462-2920.2009.02051.x.
6. Quast, C. *et al.* The SILVA ribosomal RNA gene database project: Improved data processing and web-based tools. *Nucleic Acids Res* (2013) doi:10.1093/nar/gks1219.
7. Westcott, S. L. & Schloss, P. D. OptiClust, an Improved Method for Assigning Amplicon-Based Sequence Data to Operational Taxonomic Units. *mSphere* **2**, (2017).
8. Guillou, L. *et al.* The Protist Ribosomal Reference database (PR2): A catalog of unicellular eukaryote Small Sub-Unit rRNA sequences with curated taxonomy. *Nucleic Acids Res* **41**, 597–604 (2013).
9. Rohwer, R. R., Hamilton, J. J., Newton, R. J., & McMahon, K. D. (2018). TaxAss: leveraging a custom freshwater database achieves fine-scale taxonomic resolution. Msphere, 3(5), 10-1128.
10. Hammer, U. T. (1986). Saline lake ecosystems of the world (Vol. 59). Springer Science & Business Media.
11. Bardou, P., Mariette, J., Escudié, F., Djemiel, C. & Klopp, C. SOFTWARE Open Access jvenn: an interactive Venn diagram viewer. *BMC Bioinformatics* **15**, 1–7 (2014).
